# Neuroprotective Effects of Curcumin in Methamphetamine-Induced Toxicity

**DOI:** 10.3390/molecules26092493

**Published:** 2021-04-24

**Authors:** Larisa Ryskalin, Stefano Puglisi-Allegra, Gloria Lazzeri, Francesca Biagioni, Carla L. Busceti, Linda Balestrini, Andrea Fornasiero, Stefano Leone, Elena Pompili, Michela Ferrucci, Francesco Fornai

**Affiliations:** 1 Department of Translational Research and New Technologies in Medicine and Surgery, University of Pisa, Via Roma 55, 56126 Pisa, Italy; larisa.ryskalin@unipi.it (L.R.); gloria.lazzeri@unipi.it (G.L.); michela.ferrucci@unipi.it (M.F.); 2 Istituto di Ricovero e Cura a Carattere Scientifico (I.R.C.C.S.) Neuromed, Via Atinense 18, 86077 Pozzilli, Italy; stefano.puglisiallegra@neuromed.it (S.P.-A.); francesca.biagioni@neuromed.it (F.B.); carla.busceti@neuromed.it (C.L.B.); 3 Aliveda Laboratory S.r.l., Viale Karol Wojtyla, 19, Località Pian di Laura, 56042 Pisa, Italy; l.balestrini@laboratorialiveda.com (L.B.); a.fornasiero@laboratorialiveda.com (A.F.); 4 Department of Biology, Roma Tre University, Viale Guglielmo Marconi 446, 00146 Rome, Italy; stefano.leone@uniroma3.it; 5 Department of Anatomy, Histology, Forensic Medicine and Orthopedics, Sapienza University of Rome, Via A. Borelli 50, 00161 Rome, Italy; elena.pompili@uniroma1.it

**Keywords:** *Curcuma longa*, natural polyphenol, drug of abuse, autophagy, α-synuclein, LC3, PC12 cells

## Abstract

Curcumin (CUR), a natural polyphenol extracted from rhizome of the *Curcuma longa L*, has received great attention for its multiple potential health benefits as well as disease prevention. For instance, CUR protects against toxic agents acting on the human body, including the nervous system. In detail, CUR possesses, among others, strong effects as an autophagy activator. The present study indicates that CUR counteracts methamphetamine (METH) toxicity. Such a drug of abuse is toxic by disturbing the autophagy machinery. We profited from an unbiased, low variable cell context by using rat pheochromocytoma PC12 cell line. In such a system, a strong protection was exerted by CUR against METH toxicity. This was associated with increased autophagy flux, merging of autophagosomes with lysosomes and replenishment of autophagy vacuoles with LC3, which instead is moved out from the vacuoles by METH. This is expected to enable the autophagy machinery. In fact, while in METH-treated cells the autophagy substrates α-synuclein accumulates in the cytosol, CUR speeds up α-synuclein clearance. Under the effects of CUR LC3 penetrate in autophagy vacuoles to commit them to cell clearance and promotes the autophagy flux. The present data provide evidence that CUR counteracts the neurotoxic effects induced by METH by promoting autophagy.

## 1. Introduction

In recent years, curcumin (CUR) has received great attention for its multiple potential health benefits as well as disease prevention. CUR has been explored for its multiple biological activities mostly focusing on autophagy activation [1,2,3,4,5,6], which is considered to be relevant to counteract various toxicants and disease conditions [7,8,9,10,11]. In the present manuscript we considered the potential protection exerted by CUR against methamphetamine (METH), a drug of abuse, which produces a number of neurotoxic effects in the mammalian brain including humans. We specifically challenged the protective efficacy of CUR on METH-induced toxicity based on the molecular events triggered by METH, which consist in profound alterations in the autophagy machinery.

METH intake produces toxicity in various organs including the central nervous system (CNS). For instance, METH damages nigrostriatal neurons, thereby increasing the risk of developing Parkinson’s disease (PD) in humans [12,13,14]. The intake of METH finally leads to autophagy overload. The main molecular mechanisms, which mediate METH toxicity in catecholamine-containing neurons are the following: (i) METH blocks the catecholamine uptake; (ii) it reverts the plasma membrane catecholamine (dopamine/norepinephrine) transporter (DAT/NET); (iii) it displaces the vesicular monoamine transporter type-2 (VMAT-2); (iv) it occludes the proton gradient within norepinephrine (NE)/dopamine (DA)-storing vesicles. In this way, a high amount of cytosolic NE/DA accumulates, which cannot be properly metabolized by intracellular monoamine oxidase (MAO) type A (MAO-A), since METH is also a competitive inhibitor for MAO-A (with a 10-fold higher affinity than MAO-B). In these conditions catecholamine massively invade the axo/cytoplasm. Such a severe cytosolic increase is harmful [15,16]. Altogether, catecholamine and their metabolites are highly reactive [15,17,18,19], leading to a damage to protein folding [20], which requires effective autophagy to be neutralized. Such a harmful effect of DA was magnificently demonstrated in the pioneer study by Conway et al. [21]. The authors demonstrated that, in a simple unbiased system in vitro, oxidized DA binds the protein α-synuclein, thus fostering its misfolding and oligomerization. This effect, as commented later by Sulzer, stabilizes a bad situation [22], meaning that protein misfolding is fostered by oxidized DA. In fact, this was demonstrated a few years later in vivo and in vitro [23] and confirmed later on in human patients ex vivo [24]. The cargo of misfolded protein produced by METH recruits and overwhelms the autophagy machinery, which is evident in METH intoxicated cells. Thus, in the present study we purposefully chose METH to challenge the pro-autophagic activity of CUR. The ideal biological setting to test such an interaction should include cells able to produce both catecholamines targeted by METH (DA and NE). This is represented by PC12 cell line where the storage of DA is very low making it more susceptible to cluster autophagy substrates [25]. In such a system we profited from an unbiased, low variable context to better dissect the molecular events fostered by METH and eventually counteracted by combined CUR administration. In this experimental setting, we measured CUR-induced modulation of METH-induced cell death, phenotype shift, α-synuclein aggregation, autophagy flux, autophagy proteins, merging of autophagosomes with lysosomes, and the compartmentalization of LC3 within autophagosomes. Apoptosis and slow necrosis, the latter due to inadequate autophagy machinery, where documented as well.

## 2. Results

In line with this experimental planning, at first, we characterized the safety and dose-response effects of CUR on PC12 cells to proficiently plan the doses to challenge METH-induced toxicity. Four CUR doses were selected ranging from 0.1 μM up to 50 μM. To better characterize and validate cell viability, we combined four different approaches. Namely, haematoxylin and eosin (H&E), WST-1 assay, and death specific assay such as Trypan blue (TB), to extend the analysis for the first time in such a cell line to Fluoro-Jade B (FJB) under this validated highly controlled experimental conditions. In this way, we were aiming to select the doses of CUR to challenge METH toxicity and autophagy engagement.

### 2.1. Effects of CUR on PC12 Cell Viability

**Figure 1:**
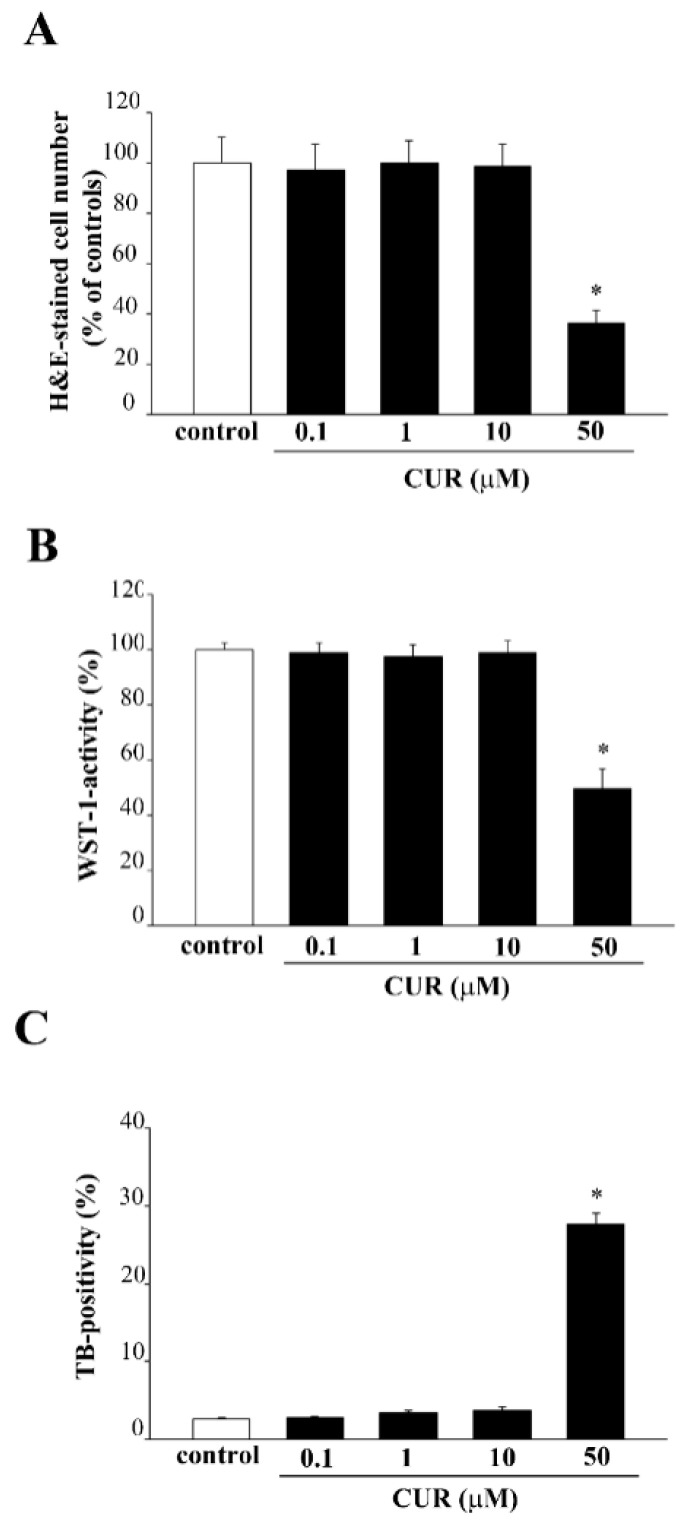
Effects of CUR on PC12 cell survival. PC12 cells were treated for 72 h with increasing doses of CUR (0.1 μM, 1 μM, 10 μM and 50 μM). Then, cell viability was measured both in control and CUR-treated cells following (**A**) H&E staining, (**B**) WST-1 assay and (**C**) TB staining. * *p* ≤ 0.05 compared with control.

As a first experimental step, we evaluated the effects of four doses of CUR (0.1 μM, 1 μM, 10 μM and 50 μM) on PC12 cell survival. As reported in Figure 1, CUR between the dose of 0.1 μM and 10 μM did not alter cell survival, when analyzed with H&E staining (Figure 1A), WST-1 assay (Figure 1B) and TB staining (Figure 1C). Only the highest dose of CUR administered in the present study (50 μM) was associated with a decrease in cell viability, as assessed through three different methods (Figure 1A–C).

These three methods are routinely used to detect cell viability on the basis of different criteria. In particular, H&E reveals the cells which, despite being subjected to a toxic insult, still preserve the ability to adhere to the slide. TB staining is a dying exclusion technique which allows to discriminate between damaged and intact cells. In detail, TB penetrates within those cells which lost the integrity of the plasma membrane, whereas it is excluded by those cells which possess an intact cell membrane. WST-1 assay is based on the cleavage of the tetrazolium salt WST-1 to formazan by cellular mitochondrial dehydrogenases. The amount of formazan dye product is correlated with the activity of the mitochondrial dehydrogenases, thus providing an indirect quantification of viable cells.

**Figure 2:**
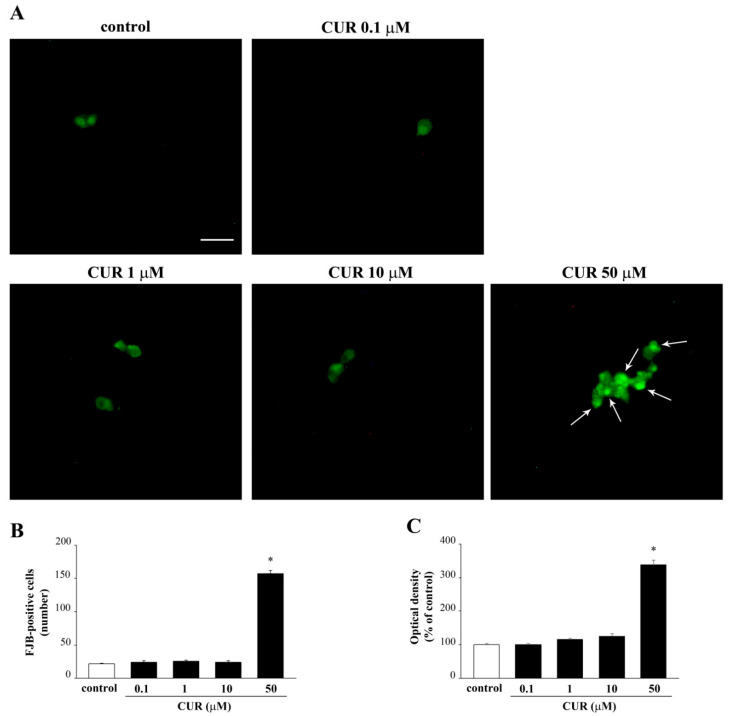
FJB-responsive PC12 cells after increasing doses of CUR. (**A**) Representative pictures of FJB-stained PC12 cells after CUR administration ranging from 0.1 μM up to 50 μM for 72 h. The graphs (**B**,**C**) report the number and the intensity of FJB fluorescent cells, respectively. Arrows indicate FJB intensely positive cells. * *p* ≤ 0.05 compared with control. Scale bar = 17.5 μM.

Data obtained with these three methods were quite convergent and they were reproduced by a fourth methodological approach used here for the first time in vitro to detect toxicity. This consists of FJB staining (Figure 2A), which provided consistent results with other methods for detecting toxicity. FJB is a fluorescein derivative used for histological staining of degenerating neurons [26]. FJB binds to molecules with multiple positive charges such as polyamines, including spermidine, putrescine, and cadaverine, which are released by dying cells [26]. FJB confirms that only the highest dose of CUR (50 μM) is toxic. This is shown by an increase both in the number and intensity of FJB fluorescent cells, as reported in the graphs (Figure 2B,C, respectively).

This experimental step was needed in order to ascertain the highest CUR dose usable without producing toxicity. It is remarkable that, the chance provided by the FJB method applies both to the number of positive cells (Figure 2B) and to the intensity of their fluorescence (Figure 2C). These increase concomitantly and to a similar extent, consistently with other procedures. The internal validation provided by the consistency of data along four different procedures makes FJB a novel suitable approach to detect METH toxicity in vitro.

We ruled out the frankly toxicant dose and we used the lower doses of CUR (0.1 μM, 1 μM, and 10 μM). Under the effects of these doses we tested the time-dependency of effects produced by CUR alone (from 24 h up to 168 h or 1 week; see Supplementary Figure S1, showing representatively the results for H&E and WST-1). Since the effects were steady and clearly established at 72 h, we selected such a time interval to analyze potential rough phenotypic changes.

### 2.2. Apoptotic Effects of CUR

**Figure 3:**
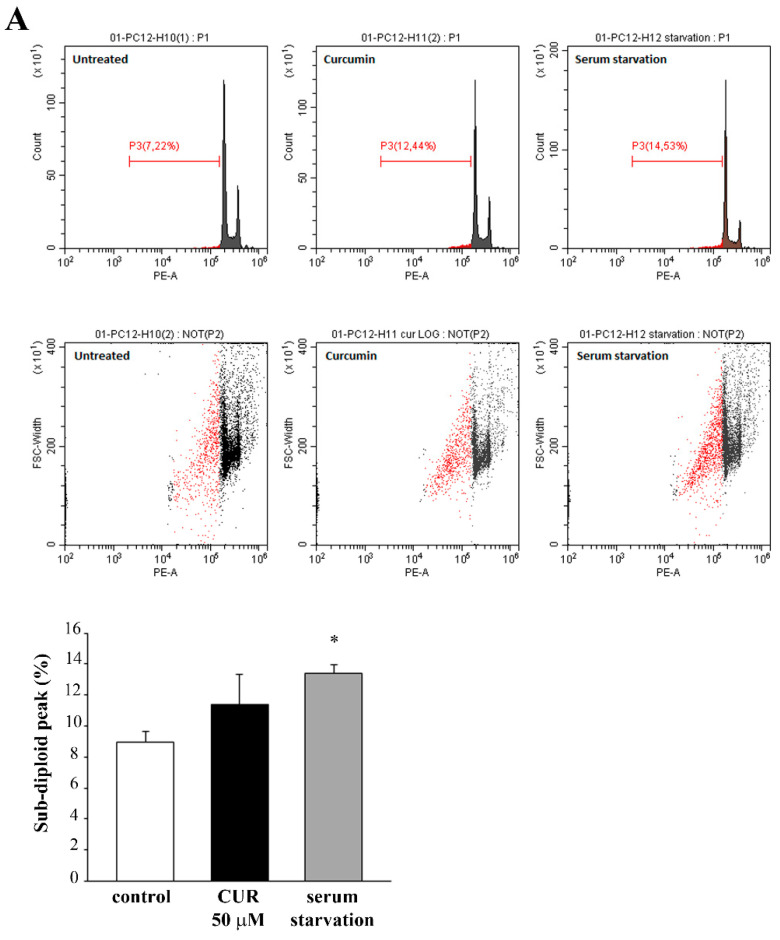

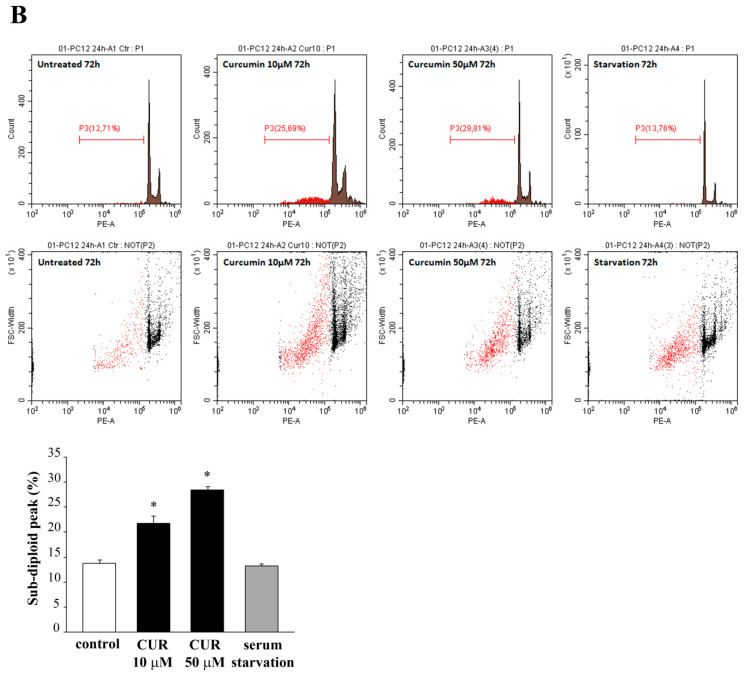
CUR induces apoptosis. Representative flow cytometry profiles and the related histogram reporting the sub-G1 peak after treatment with CUR for (**A**) 24 h (50 μM) and (**B**) 72 h (10 μM and 50 μM), compared with untreated control cells. Data related to serum starvation were shown as positive control. * *p* ≤ 0.05 compared with control.

In additional experiments we investigated whether CUR-induced cytotoxicity was mediated via an apoptotic mechanism. In detail, we evaluated the sub-G1 peak through flow cytometry for a time-course study in PC12 cells treated with CUR at 50 μM, for 24 h and 72 h. We chose this dose of CUR since it produces significant cell loss. Flow cytometry data with PI staining shows that CUR at 50 μM induces apoptosis peaking at 72 h, as witnessed by the sub-G1 peak (Figure 3).

**Figure 4:**
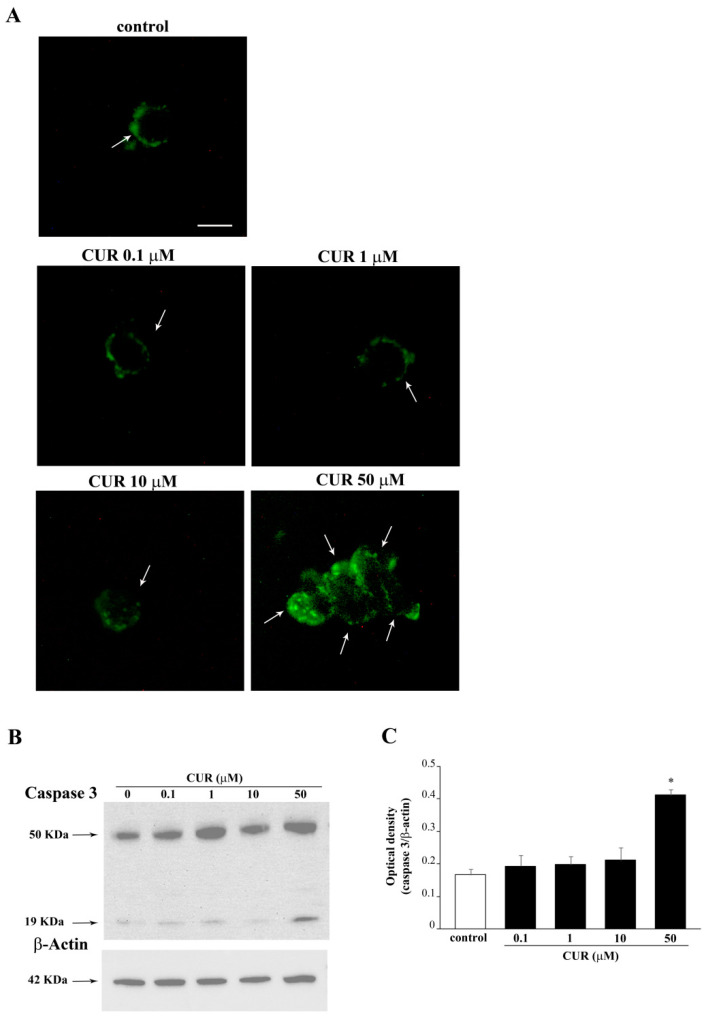
CUR 50 μM increases the cleaved form of caspase 3. (**A**) Representative pictures showing caspase 3-immunofluorescent PC12 cells after CUR administration ranging from 0.1 μM up to 50 μM for 72 h. Immunoblotting for caspase 3 (**B**) and the related graph reporting the optical density (**C**). The primary antibody recognizes the cleaved form of caspase 3. Blot at 50 kDa represents non-specific caspase 3 substrates which are also detected at WB. Arrows indicate caspase 3 positive cells. * *p* ≤ 0.05 compared with control. Scale bar = 7.3 μM.

In addition, only at 72 h a challenge with a lower dose of CUR at 10 μM was carried out. This was tested to document whether a slight apoptotic effect could be detected even in the absence of significant cell loss. In fact, CUR 10 μM administered for 72 h produces a slight evidence for apoptosis; however, this was evident at flow cytometry (Figure 3B), although this could not be detected by other methods, first of all by TEM. Instead, the frank apoptosis detected for the CUR dose of 50 μM was confirmed by WB and immunofluorescence for the apoptotic marker caspase 3 (Figure 4).

**Figure 5:**
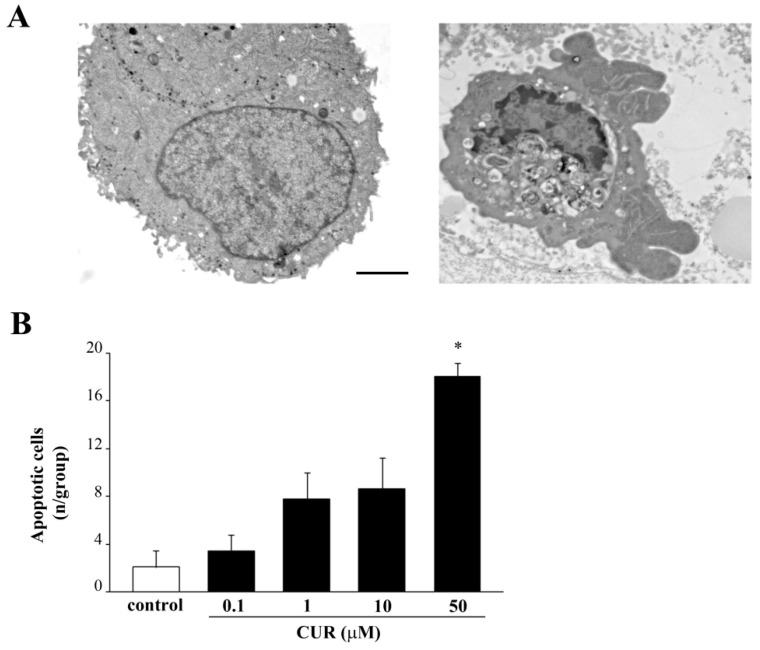
CUR 50 μM increases the number of apoptotic cells. (**A**) Representative micrographs of PC12 cells of control and following CUR 50 μM for 72 h. (**B**) The histogram reports the number of apoptotic PC12 cells counted at TEM after CUR administration ranging from 0.1 μM up to 50 μM for 72 h. * *p* ≤ 0.05 compared with control. Scale bar = 1 μM.

The primary antibody used in both procedures specifically binds small caspase 3 fragments, which are produced following caspase 3 activation, while it does not recognize the inactivated full-length form of caspase 3. In fact, activated caspase 3 mostly occurs only when the highest dose of CUR is administered. In contrast, we cannot find activated caspase 3 in cells treated with CUR 10 μM (Figure 4), which still, produces a percentage of cell at sub-G1 detected at flow cytometry (Figure 3B). Gold- standard plain transmission electron microscopy (TEM) provided the most solid evidence to quantify apoptotic cells produced following CUR by detecting specific morphological changes (i.e., condensed chromatin, nuclear bodies) which indicate an ongoing apoptotic process. Count of frankly apoptotic cells following CUR at 0.1 μM, 1 μM, 10 μM, and 50 μM demonstrate that only the highest dose of CUR produces a significant increase in the number of apoptotic cells, as shown by representative micrographs (Figure 5A,B).

### 2.3. CUR-Induced Phenotypic Switch

**Figure 6:**
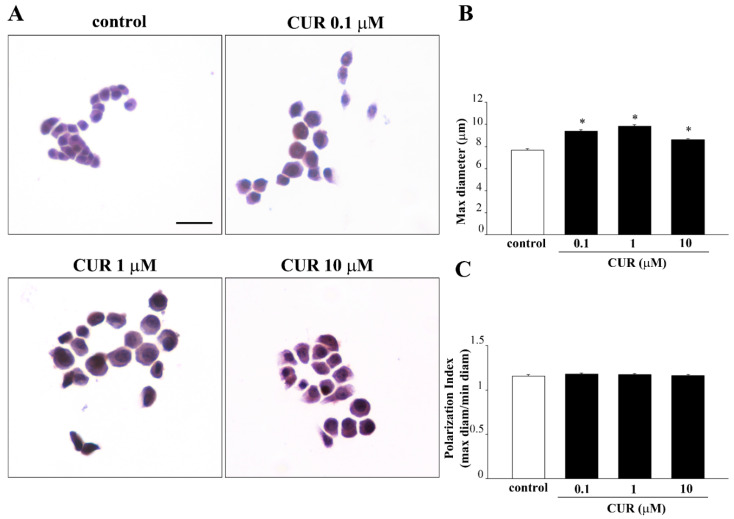
CUR treatment induces morphological changes in PC12 cells. (**A**) Representative pictures of H&E-stained PC12 cells after CUR administration ranging from 0.1 μM up to 10 μM for 72 h. The increased size of the cell body in CUR-treated cells is evident, as reported in the graph (**B**). Histogram (**C**) shows the Polarization Index as the ratio between the maximum and minimum cell diameter. * *p* ≤ 0.05 compared with control. Scale bar = 19.4 μM.

Exposure to increasing doses of CUR for 72 h produces morphological changes in PC12 cells. In fact, while control PC12 cells feature a small and round cell body, CUR administration (ranging from 0.1 μM up to 10 μM for 72 h) produces a significant increase in cell size compared with controls (Figure 6A). In detail, the maximum diameters of PC12 cells exposed to 0.1, 1 and 10 μM of CUR were 9.41 ± 0.14, 9.82 ± 0.15 and 8.60 ± 0.12 µm, respectively and were on average 1.2-fold higher than the maximum diameter of control cells (7.68 ± 0.12 µm, Figure 6B). However, CUR-induced increase in cell size did not lead to a polarization of the cell body. In fact, as shown in Figure 6C, CUR did not affect the Polarization Index, calculated as the ratio between the maximum and minimum cell diameter, which remains unchanged.

### 2.4. Dose-Response Curve of METH on PC12 Cell Viability

**Figure 7:**
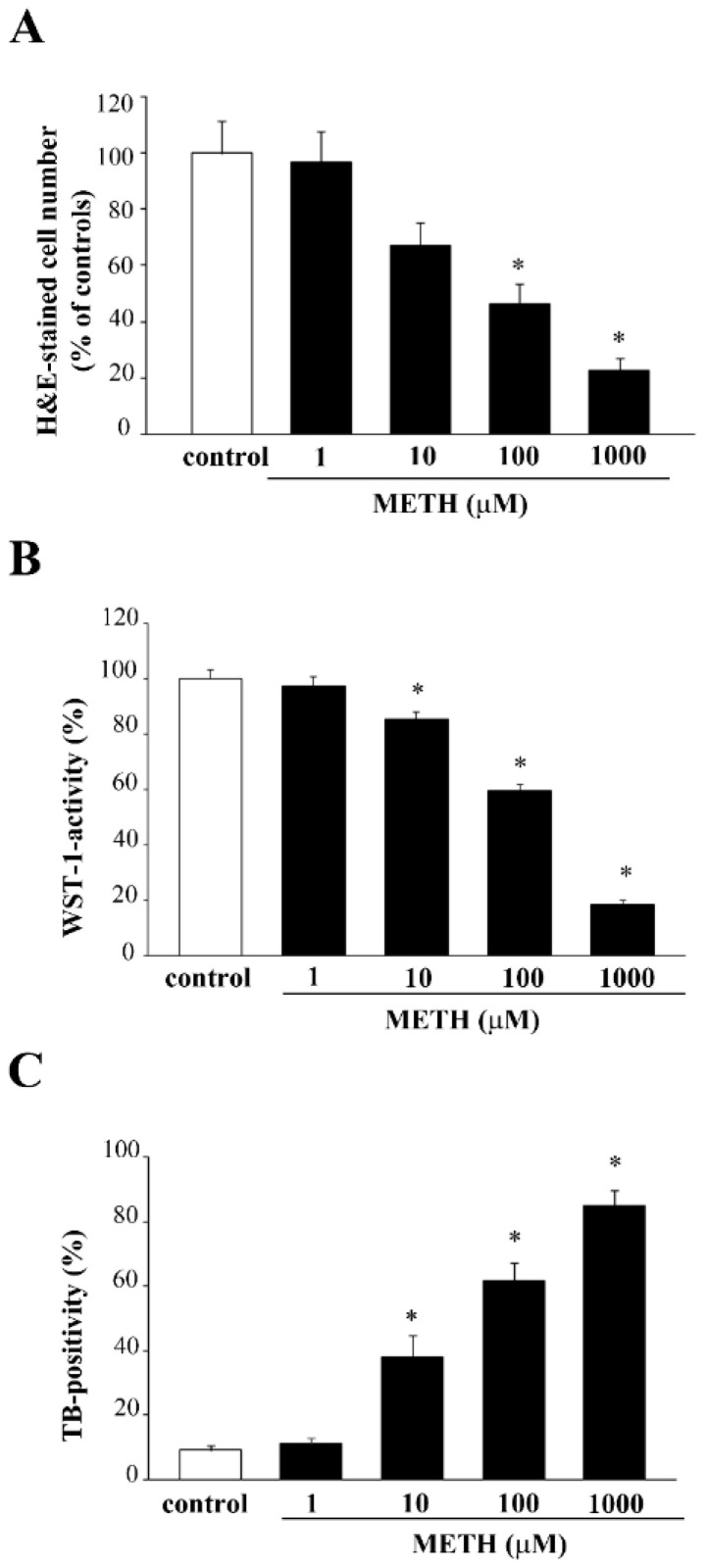
METH dose-dependently affects PC12 cell survival. PC12 cell survival decreases after increasing doses of METH (1 μM, 10 μM, 100 μM, 1000 μM) for 72 h dose-dependently. Cell viability was evaluated with (**A**) H&E staining, (**B**) WST-1 activity (**C**) TB-positivity. * *p* ≤ 0.05 compared with control.

**Figure 8:**
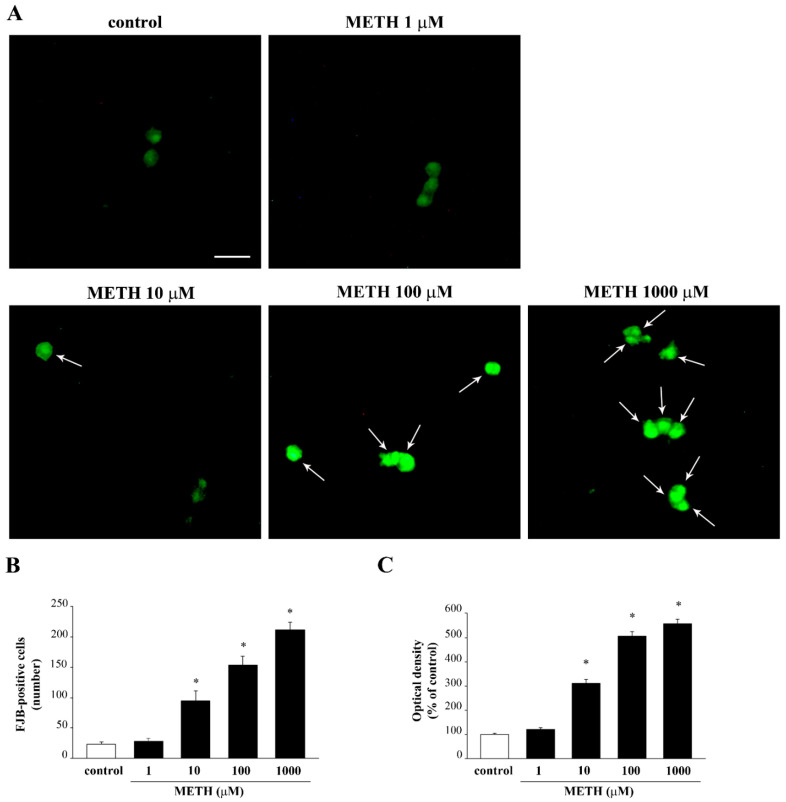
Dose-dependent Fluoro-Jade B-responsiveness of PC12 cells after METH exposure. (**A**) Representative pictures of FJB-stained PC12 cells after METH administration ranging from 1 μM up to 1000 μM for 72 h. The graphs shows the number (**B**) and intensity (**C**) of FJB fluorescent cells (arrows). * *p* ≤ 0.05 compared with control. Scale bar = 17.1 μM.

Based on a number of previous studies carried out by the research team in vitro with METH, we selected a specific time window ranging from 24 h up to 168 h to select the best time interval for METH continuous exposure. In fact, each specific phenomenon under investigation occurs in this time interval. The specific time we refer in the article body was selected at 72 h (Supplementary Figure S2) since this corresponds to the time needed to develop fully METH toxicity in a wide range of doses. The toxic plateau was achieved at 72 h. Thus, in the body of the article the effects produced by four METH doses ranging from 1 μM up to 1000 μM on PC12 cells at 72 h are reported. As tested for CUR, cell viability (Figure 7) was measured following four procedures: H&E (Figure 7A), WST-1 assay (Figure 7B), TB staining (Figure 7C) and FJB (Figure 8). The loss in cell viability was significant at 10 μM according to WST-1, TB and FJB assay, while H&E staining did not provide significant effects up to 100 μM METH dose. This is likely to be due to the staining sensitivity, which provides positive data for dying or dead cells with TB and FJB. This is also the case of WST-1, which may be impaired when cell metabolism is strongly altered, despite the cell is still alive. In contrast, H&E staining does not provide direct measurement of cell death albeit disclosing fine cell alterations.

As commented above, FJB staining (Figure 8A) provides evidence for a significant loss in cell viability from METH 10 μM up to 1000 μM (both when counting the number of positive cells and when measuring the intensity of fluorescence, as reported in the graphs, Figure 8B,C, respectively).

As mentioned for the toxic dose of CUR, to our knowledge this is the first time that FJB is applied to PC12 cell culture [27,28]. Furthermore, this is the first time that such a technique is used in vitro to document METH toxicity. Therefore, the present results also work as an internal validation of such a procedure to reliably assess cell damage following METH in vitro. This is confirmed by the overlapping between FJB data and other methods used here to assess the viability in this neural crest-derived cell line. This method provides data, which are consistent with other classic, well validated methods [29,30,31,32] and it provides vivid images, which are representative of cell toxicity (considering the amount and intensity of fluorescence).

### 2.5. Phenotypic Effects of METH

**Figure 9:**
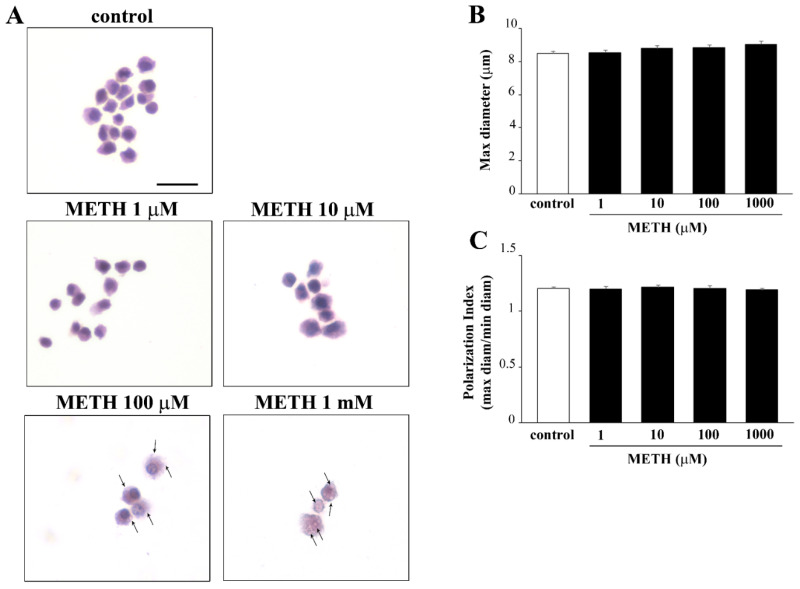
Dose-dependent METH-induced morphological changes in PC12 cells. (**A**) Representative pictures of H&E-stained PC12 cells after METH administration. Cytosolic pale vacuoles (arrows). (**B**) Graph reports the maximum cell diameter and the Polarization Index (**C**). Scale bar = 24.3 μM.

This paragraph, despite providing gross information, serves to better clarify frank cell alterations documented through H&E following METH. This study documents the occurrence of pale cytosolic enlarged areas (Figure 9), which are reminiscent of altered spared cells as expected following a neurotoxin, which causes vacuolar degeneration due to autophagy disruption. Nonetheless, for the low METH doses (1 μM and 10 μM), we failed to document changes in diameter and polarization, which suggests how phenotype varies according to the onset of toxicity.

### 2.6. Dose-Dependent CUR-Induced Protection against METH Toxicity

**Figure 10:**
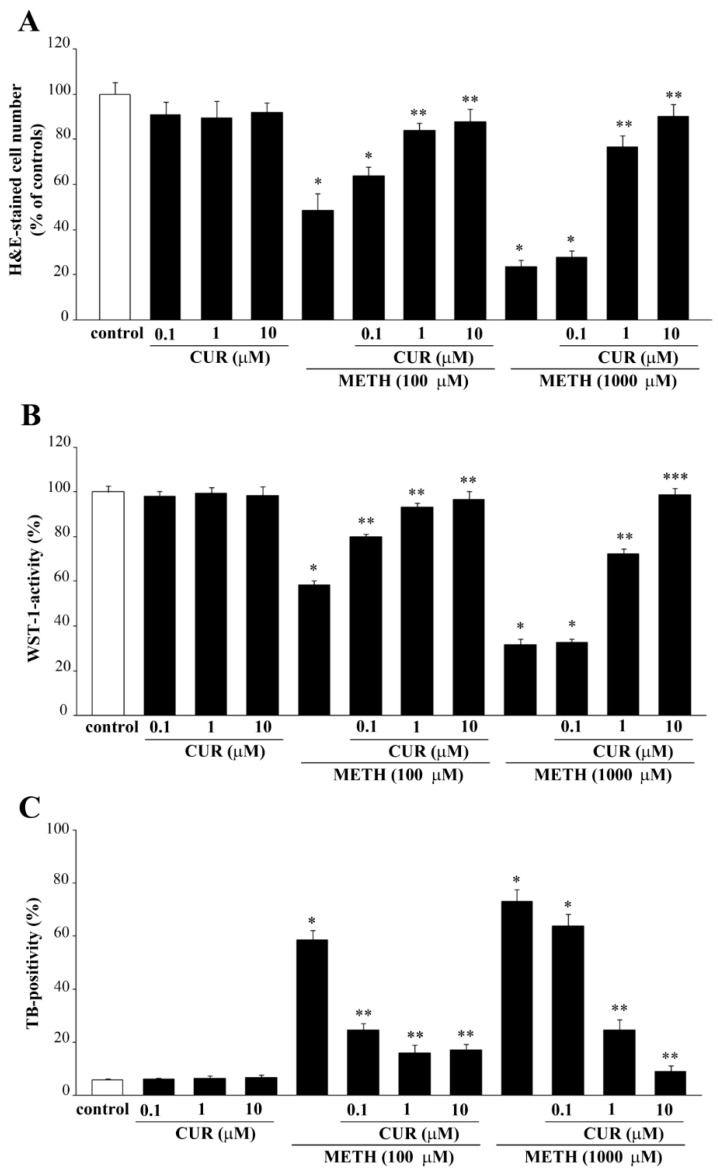
CUR prevents METH-induced toxicity in PC12 cells. After administration of METH, alone or in combination with CUR for 72 h, PC12 cell survival was assessed through (**A**) H&E staining, (**B**) WST-1 assay and (**C**) TB staining. * *p* ≤ 0.05 compared with control; ** *p* ≤ 0.05 compared with METH; *** *p* ≤ 0.05 compared with METH 1000 μM + CUR 1 μM.

Since the dose of METH of 100 μM produces a moderate toxicity and the dose of 1000 μM provides the highest (about 80%) cell loss, these two doses of METH were selected for combined treatment with three CUR doses. When CUR was administered at the dose of 0.1 μM we failed to document a significant protection against the highest dose of METH (1000 μM), while a significant rescue of cell viability was documented for the dose of METH of 100 μM (Figure 10A–C). When CUR was administered at the dose of 1 μM and 10 μM we observed a protection against both METH 100 μM and 1000 μM (Figure 10A–C). Thus, CUR provides full protective efficacy already at the dose of 1 μM when METH was administered at the dose of 100 μM; however, to achieve a full protection against METH 1000 μM the highest dose of CUR (10 μM) was needed (Figure 10A–C).

**Figure 11:**
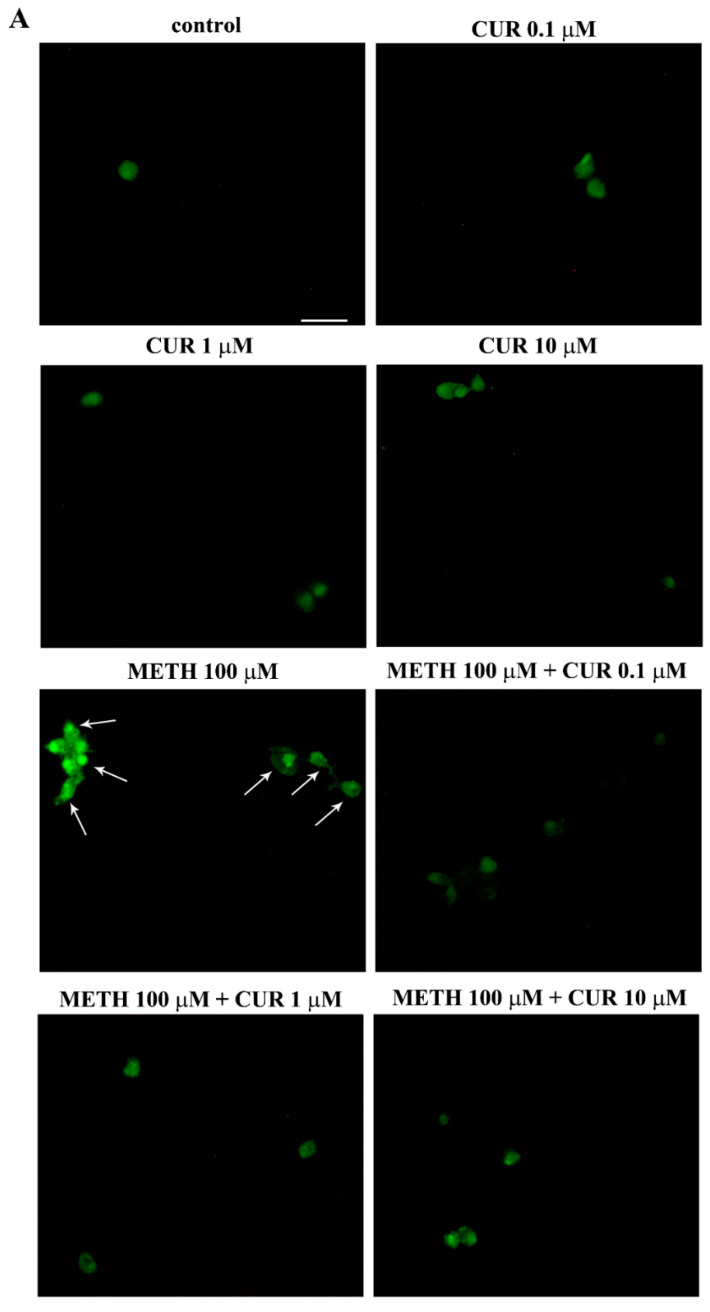

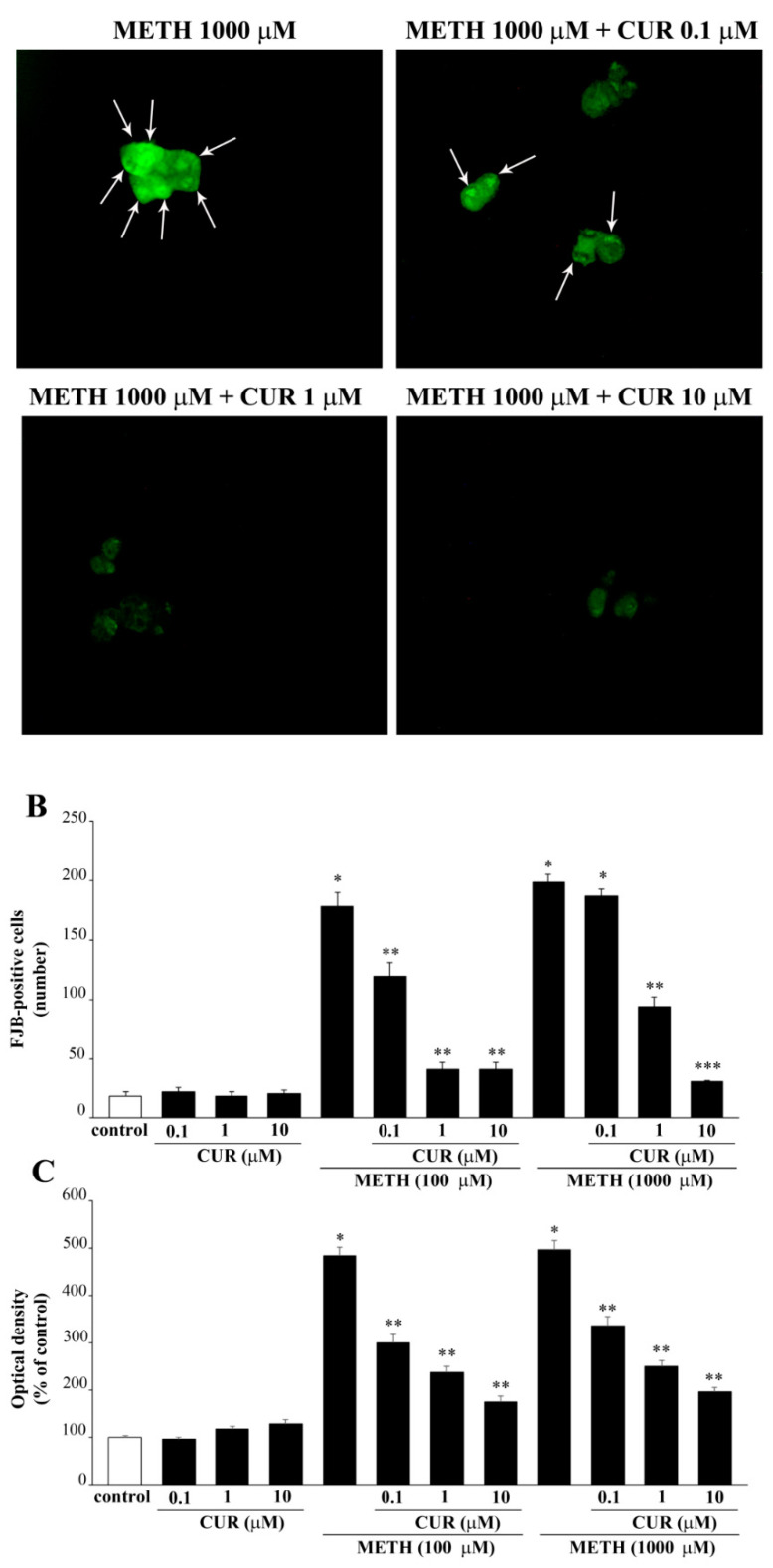
CUR decreases METH-induced FJB-responsiveness in PC12 cells. (**A**) Representative pictures of FJB-stained PC12 cells after various treatments The graphs (**B**,**C**) report the number and the intensity of FJB fluorescent cells, respectively. Arrows indicate FJB intensely positive cells. * *p* ≤ 0.05 compared with control; ** *p* ≤ 0.05 compared with METH 100 μM; *** *p* ≤ 0.05 compared with METH 1000 μM + CUR 1 μM. Scale bar = 17.3 μM.

Again, these data are in line with those obtained after FJB staining (Figure 11A), which confirm how pre-treatment with CUR decreases METH-induced toxicity in PC12 cells. In fact, combined treatments with METH 100 μM and CUR 0.1 μM, 1 μM or 10 μM significantly decreased both the number and the intensity of FJB fluorescent cells compared to METH-treated cells, which is evident in representative images of FJB staining (Figure 11A) and it is also reported in the graphs (Figure 11B,C, respectively). In contrast, only the highest doses of CUR (1 μM and 10 μM, were able to prevent the increase in the number and intensity of FJB-positive PC12 cells induced by METH 1000 μM (Figure 11A–C).

### 2.7. Reversal by CUR of METH-Induced Cellular Alterations within Spared Cells

**Figure 12:**
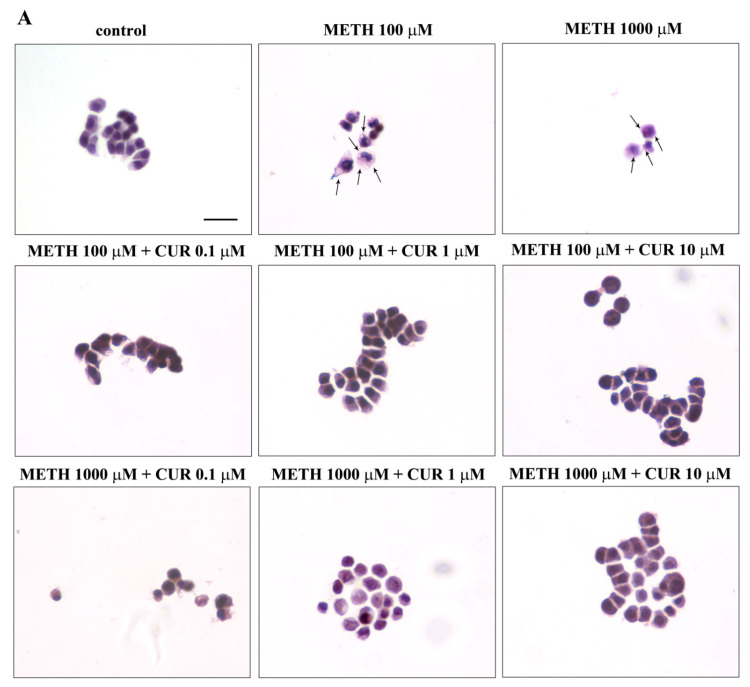

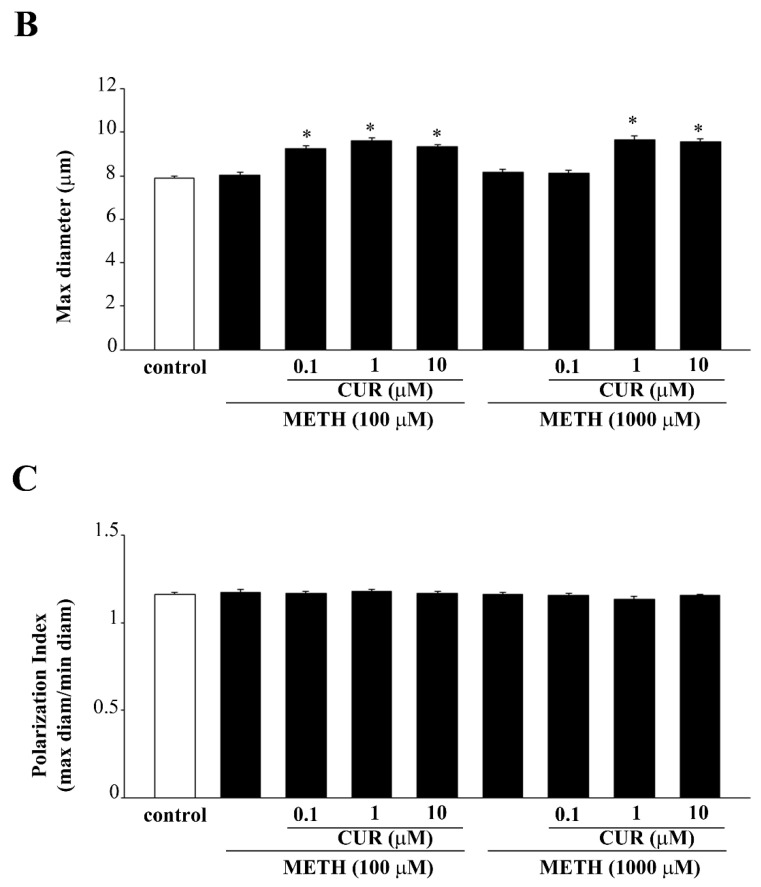
CUR prevents METH-induced morphological alterations. (**A**) Representative pictures of H&E-stained PC12 cells after various treatments. Cytosolic vacuoles occurs in METH-treated cells (arrows). (**B**) Maximum cell diameter and (**C**) Polarization index are reported. * *p* < 0.05 compared with control. Scale bar = 19.5 μM.

We chose such a title to avoid considering the effect of combined treatments as a sort of phenotypic shift but rather interpreting these changes as a rescue of cell alterations produced by METH under CUR modulation. In fact, the dose response curve for such an effect reproduces what described for CUR-induced modulation of METH toxicity described at previous paragraph. However, when measuring cell alterations, the rescuing activity of CUR was better tailored by the specific doses administered to the cell. In fact, the dose of 1 μM and 10 μM CUR were both fully protective against cell loss, when observed in their fine light morphology by classic H&E staining. The dose of 1 μM, despite granting cell survival, did not revert most of cell alterations, pale cytosol, which were mostly erased by the dose of 10 μM of CUR (Figure 12).

### 2.8. Dose-Dependent Effects of CUR on the Expression of α-Synuclein in Control Cells and Following METH Administration

**Figure 13:**
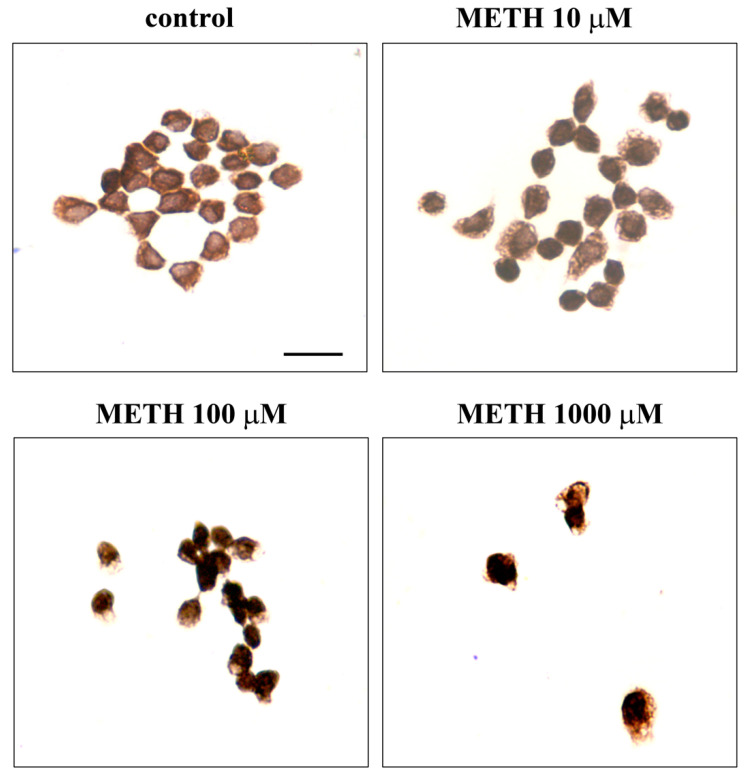
METH dose-dependently induces expression of α-synuclein. Representative immunocytochemistry for α-synuclein following METH. Scale bar = 19.2 μM.

Since α-synuclein accumulation does occur following METH administration [22,23,30,33,34] potentially contributing to METH toxicity, we wondered which sort of effects were exerted by CUR on α-synuclein levels. In detail, once the protective effects of CUR were demonstrated we wondered whether this was accompanied by a mitigation of METH-induced α-synuclein accumulation. The effects produced by various CUR doses on baseline α-synuclein in PC12 cells were documented. In fact, as we introduced in the present manuscript, such a cell line owns aberrancies which recapitulate the mechanisms of catecholamine neurotoxicity [35]. In fact, PC12 cells possess a high level of freely diffusible DA, with a low storage ability and the trend to polarize the secretory vesicles toward the plasma membrane. Such a condition is not properly healthy, in fact roughly 5% of the cell die in baseline conditions (see Figure 1, Figure 7 and Figure 10). This explains the potential occurrence of protein misfolding in control cells and the potential promoting of protein clearance by CUR even compared with control conditions. Such an issue was explored in the present study. In fact, control PC12 cells possess a considerable amount of α-synuclein, as reported in representative Figure 13.

**Figure 14:**
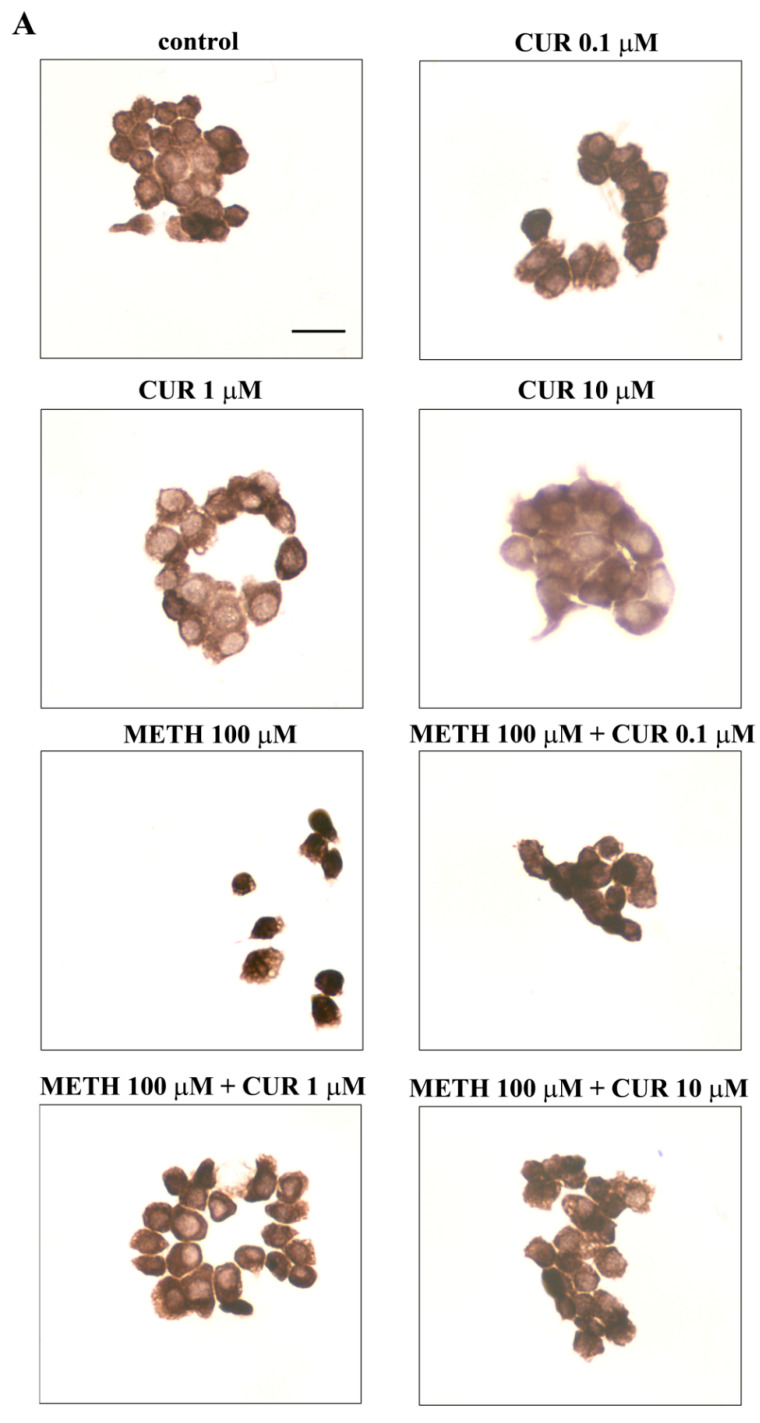

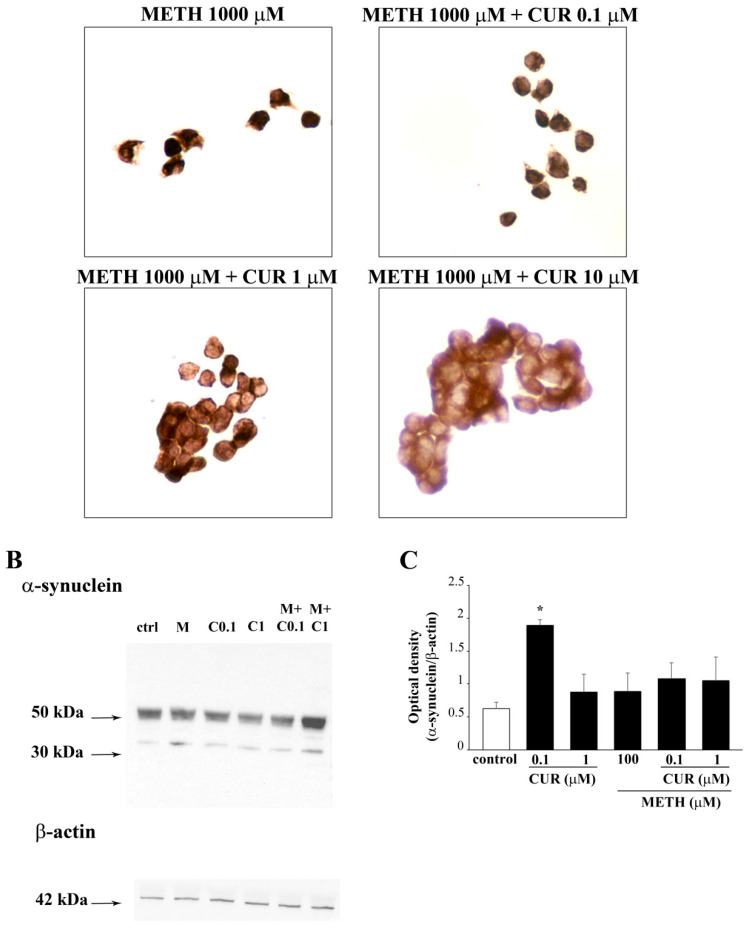
CUR mitigates the expression of α-synuclein in control and METH-treated cells. (**A**) Representative immunostaining for α-synuclein. (**B**) Representative immunoblotting for α-synuclein and β-actin in control and PC12 treated cells and the related optical densities (**C**). (Ctrl = control; M = METH 100 μM; C0.1 = CUR 0.1 μM; C1 = CUR 1 μM; M + C0.1 = METH 100 μM + CUR 0.1 μM; M + C1 = METH 100 μM + CUR 1 μM); * *p* < 0.05 compared with control. Scale bar = 16.1 μM.

As expected and previously documented [22,23,30] METH dose-dependently increases α-synuclein levels, which was detected at immunohistochemistry. In fact, when toxic doses of METH were administered, α-synuclein levels were slightly increased at METH 10 μM to reach a considerable amount for the METH dose of 100 μM, which reaches a sort of plateau, since at 1000 μM no further increment was detected (Figure 13). In contrast, when CUR was administered at three different doses (from 0.1 μM up to 10 μM), a significant, robust decrease in α-synuclein was detected both at light microscopy (Figure 14A). Despite the representative immunostaining shows that CUR may reduce baseline α-synuclein levels (Figure 14A), western blotting does not provide any noticeable effect (Figure 14B,C, likely due to the inherent limitation of such a procedure).

Remarkably, when observing the immunoblotting in control PC12 cells α-synuclein was scarce. Instead, the dimeric α-synuclein was evident along with the polymeric protein, which again highlights the peculiarity of PC12 cells (Figure 14B). When a different non catecholamine cell line (i.e., U87 MG) was challenged to validate such an aberrancy, we detected the low molecular weight monomeric α-synuclein, which does not appear in PC12 cells.

When combined METH and CUR were administered CUR provided a significant and complete reversal of METH-induced α-synuclein expression, as reported below. In detail, this effect was remarkable when considering that CUR belongs to polyphenols phytochemical and the relevance of α-synuclein accumulation in degenerative disorders. Thus, such a powerful effect here demonstrated by CUR deserves specific attention. When this was maximally expressed (already at the dose of CUR 1 μM) the amount of α-synuclein produced by the dose of 100 μM METH was roughly comparable with controls (Figure 14A). A similar potency was detected by western blotting where the CUR doses of 0.1 μM and 1μM tear down the levels of METH-induced α-synuclein back to an amount comparable with controls (Figure 14B,C). This was unexpected since it indicates that CUR erases α-synuclein accumulation produced by each dose of METH, which might possess strong implications. The magnitude of the CUR-induced suppression of METH-induced α-synuclein aggregation might per se justify the protection of CUR against METH toxicity. Nonetheless, it is likely that such a clean cut effect indeed represents the “tip of an iceberg”, since α-synuclein is removed by autophagy as much as a number of toxic misfolded proteins. If CUR activates autophagy, the removal of α-synuclein is just representative of a generalized cell clearance, which may extend to a bulk of toxic protein cargoes each potentially contributing to METH toxicity. Therefore it is tempting to probe the magnitude of this effect compared with the intensity of autophagy induction, which is expected to be produced by CUR.

**Figure 15:**
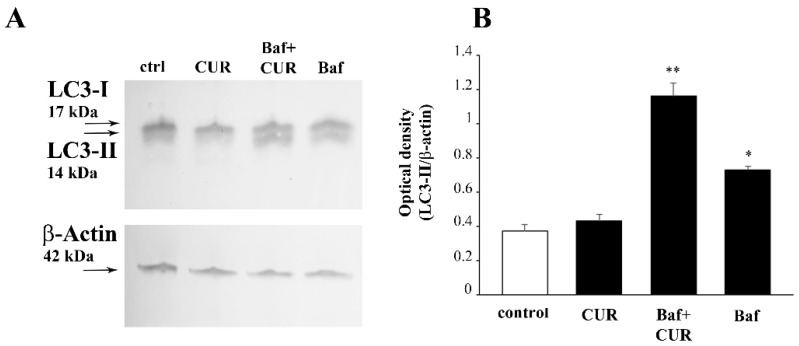
CUR induces autophagy. (**A**) WB for LC3-II levels and (**B**) related optical density graph in PC12 cells treated with CUR and the late autophagy inhibitor bafilomycin A1. Ctrl = control; Baf = bafilomycin A1 * *p* < 0.05 compared with control; ** *p* < 0.05 compared with control and Baf.

In order to measure the autophagy flux in CUR-treated cells, we administered bafilomycin A1 50 nM, a potent inhibitor of the vacuolar-type H^+^ ATPase, which results in lysosomal impairment and inhibition of the late stages of autophagy (which includes a block of LC3 degradation, [36,37]). In fact, the full length LC3 (or LC3-I) is a microtubule-associated protein which is converted in its membrane-associated isoform LC3-II when autophagy is induced. In particular, LC3-I is partially cleaved at the C-terminus to generate the isoform II, which is then conjugated to phosphatidylethanolamine just prior to its insertion onto the autophagosomal membrane. Both LC3-I and LC3-II are rather unstable, and LC3-II is rapidly degraded by lysosomal proteases once the autophagolysosome is formed [38]. Therefore, the amount of LC3-II is predictive of the autophagy status, being low when autophagy is ongoing while increasing when the late autophagy is inhibited. Accordingly, WB with a primary antibody which allows to detect both LC3-I and LC3-II indicates that in bafilomycin A1-treated cells LC3-II accumulates (Figure 15). 

Conversely, CUR 10 μM attenuates LC3-II accumulation induced by bafilomycin A1, which was added to the culture medium 3 h before the end of the CUR treatment (Figure 15). These results demonstrate that CUR at the dose of 10 μM, which provides the major protection against METH toxicity, reduces the levels of LC3-II and counteract the accumulation of LC3-II induced by bafilomycin by promoting the autophagy flux (Figure 15).

### 2.9. CUR Increases LC3 and Autophagy Vacuoles

**Figure 16:**
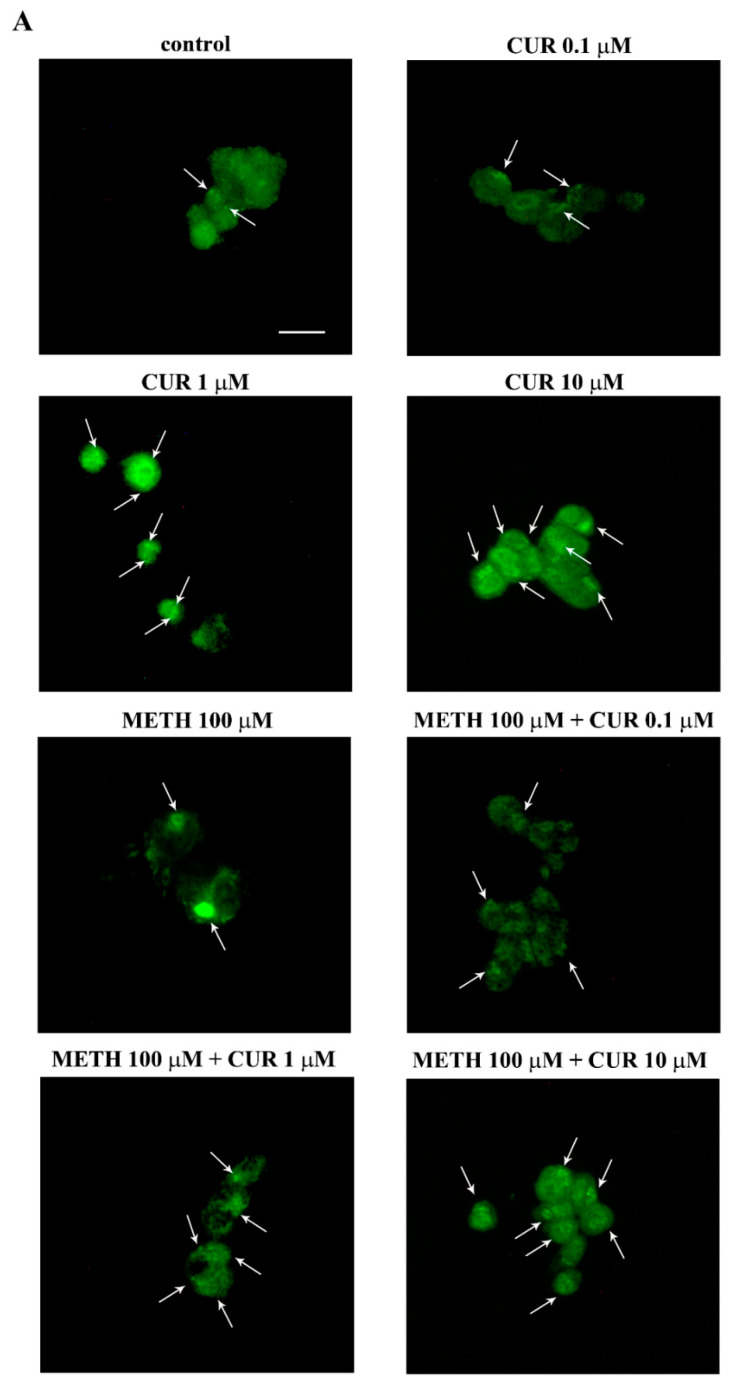

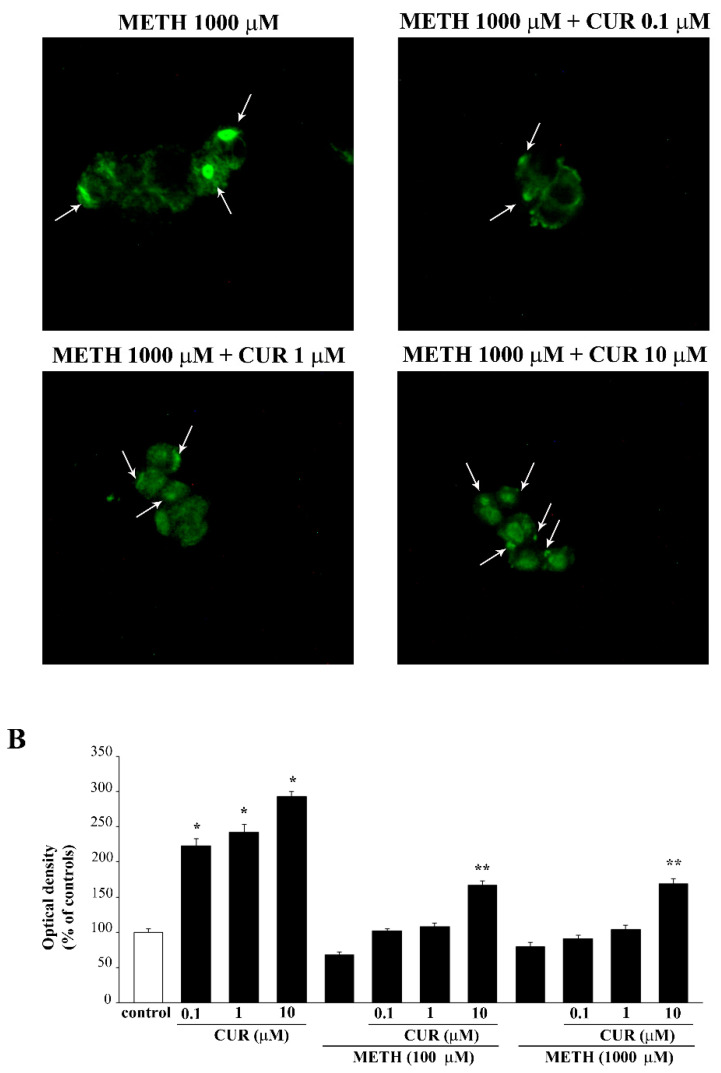
CUR increases LC3 immunofluorescence in control and METH-treated PC12 cells. (**A**) Immunofluorescence for LC3. Arrows indicate fluorescent puncta and bigger immunofluorescence agglomerates. (**B**) The graph reports the intensity of the immunofluorescence measured as optical density. * *p* < 0.05 compared with control; ** *p* < 0.05 compared with METH. Scale bar = 14.2 μM.

CUR alone provides an increase in the amount of whole cell LC3, as measured by immunofluorescence and evidenced in representative Figure 16.

CUR administration (ranging from 0.1 μM up to 10 μM) dose-dependently increases the intensity and number of immunofluorescent “puncta” compared with control cells. A similar effect is observed when CUR is administered in combination with METH. In fact, as shown in the representative pictures of Figure 16A, the big agglomerates of immunofluorescence which are produced by METH both at 100 μM and 1000 μM, were reverted in smaller “puncta” by CUR in a dose-dependent manner. This effect is mostly evident for the highest CUR doses, namely 1 μM and 10 μM.

**Figure 17:**
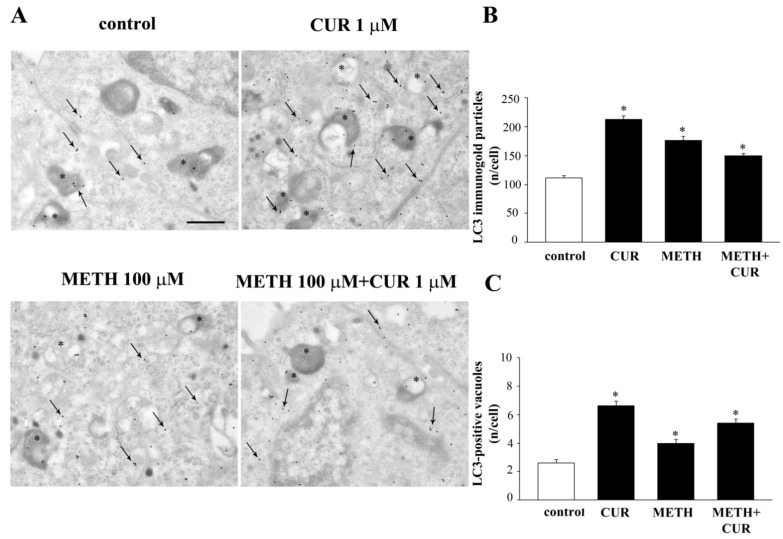
CUR increases the amount of LC3-positive vacuoles. (**A**) Representative TEM micrographs showing LC3-positive vacuoles (*) Arrows point to LC3 immuno-gold particles widespread in the cytosol. Graphs report the number of LC3-immunogold particles (**B**) and LC3-positive vacuoles (**C**) per cell. * *p* < 0.05 compared with control. Scale bar = 0.3 μm.

This robust effect is magnified when the amount of LC3 within autophagy vacuoles is counted in CUR-treated cells compared with controls, as shown by TEM (Figure 17).

These phenomena suggest that CUR behaves as a powerful autophagy activator, which apart from increasing LC3 indeed mostly commits such a molecule to the autophagy machinery. In fact, CUR at 1μM polarizes LC3 immuno-gold particles within autophagy vacuoles. Such a polarization exceeds that measured in control cells (Figure 17C).

### 2.10. CUR Counteracts METH-Induced Depolarization of LC3 from Autophagy Vacuoles

When METH (100 μM and 1000 μM) is administered despite big compartments of the cell intensely stain for LC3 (Figure 16) LC3 immunostaining does not appear to be widespread within the cytosol (Figure 16). Conversely, a homogeneous fine punctate trim is detected in LC3 immunostaining when CUR is administered either alone or in combination with METH (Figure 16). When dissected at TEM such a punctate immunofluorescence is resolved and further deciphered. In fact, the large and intense LC3 immunostaining produced by METH administration is not related to autophagy vacuoles, but it is rather an effect of depolarization of LC3 from widespread autophagy vacuoles within partial zones of the cytosol. This confirms what recently demonstrated at molecular level. In fact, METH does not freeze LC3 positive autophagy vacuoles impairing their progression, but it rather removes LC3 from the vacuoles itself. In this way, METH de-potentiates the autophagy machinery already during early step of autophagy progression. Such a de-funding of autophagy vacuoles from LC3 impairs the ability to degrade misfolded proteins and mitochondria, which undergo accumulation within intoxicated cells. The addition of CUR re-establishes the polarization of autophagy vacuoles, thus empowering back the autophagy machinery. This is just the phenomenon which is likely to produce the clearance of α-synuclein as reported above as well as other misfolded proteins and mitochondria. This suggests that CUR may rescue METH intoxicated cells mostly grounded on this specific effect. In fact, we could demonstrate a similar mechanism when METH-intoxicated cells were protected by the gold standard autophagy inducer, mTORC1-inhibitor rapamycin [38].

### 2.11. Autophagy Inhibition Occludes the Protective Effect of CUR against METH Toxicity

**Figure 18:**
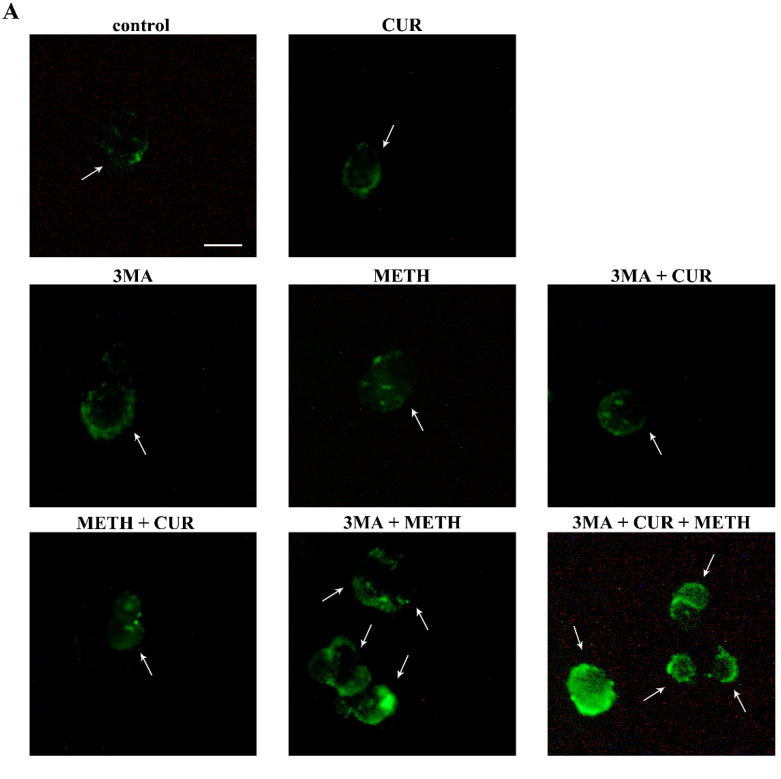

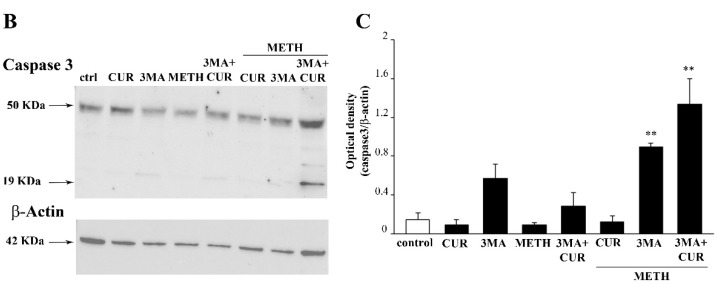
Inhibition of autophagy precipitates apoptotic cell death in METH-treated cells. (**A**) Representative pictures of caspase 3-immunofluorescent cells in PC 12 cells treated with CUR 10 μM, METH 100 μM, and the autophagy inhibitor 3MA. Immunoblotting for caspase 3 (**B**) and the related optical density graph (**C**) The primary antibody recognizes the cleaved form of caspase 3. Blot at 50 kDa represents non-specific caspase 3 substrates which are also detected at WB. Arrows indicate caspase 3 positive cells. ** *p* ≤ 0.05 compared with control and METH. Scale bar = 7.3 μM.

In order to in-depth investigate the role of autophagy in the protective effects of CUR, we used the pharmacological autophagy inhibitor 3-methyladenine (3MA). 3MA specifically blocks autophagosome formation by inhibiting class III phosphatidylinositol-3-phosphate kinase (PI3k) activity [39,40]. Autophagy inhibition in the presence of METH is detrimental for cell survival, as shown by H&E staining, WST-1 assay, and TB staining, reported in Supplementary Figures S3–S5. In detail, we demonstrated that 3MA-induced cell death was apoptotic in nature thus confirming the results obtained in our previous study [30]. In fact, the autophagy inhibitor 3MA precipitates apoptotic cell death when administered in combination with METH, and this effect is not reversed by CUR. This is shown by WB assay and representative immunofluorescence against the cleaved form of caspase 3 (Figure 18). It is remarkable that when autophagy is inhibited, the dose of CUR (10 μM) which produces the highest protection against METH was no longer able to prevent the apoptotic cell death in 3MA+METH-treated cells (Figure 18).

**Figure 19:**
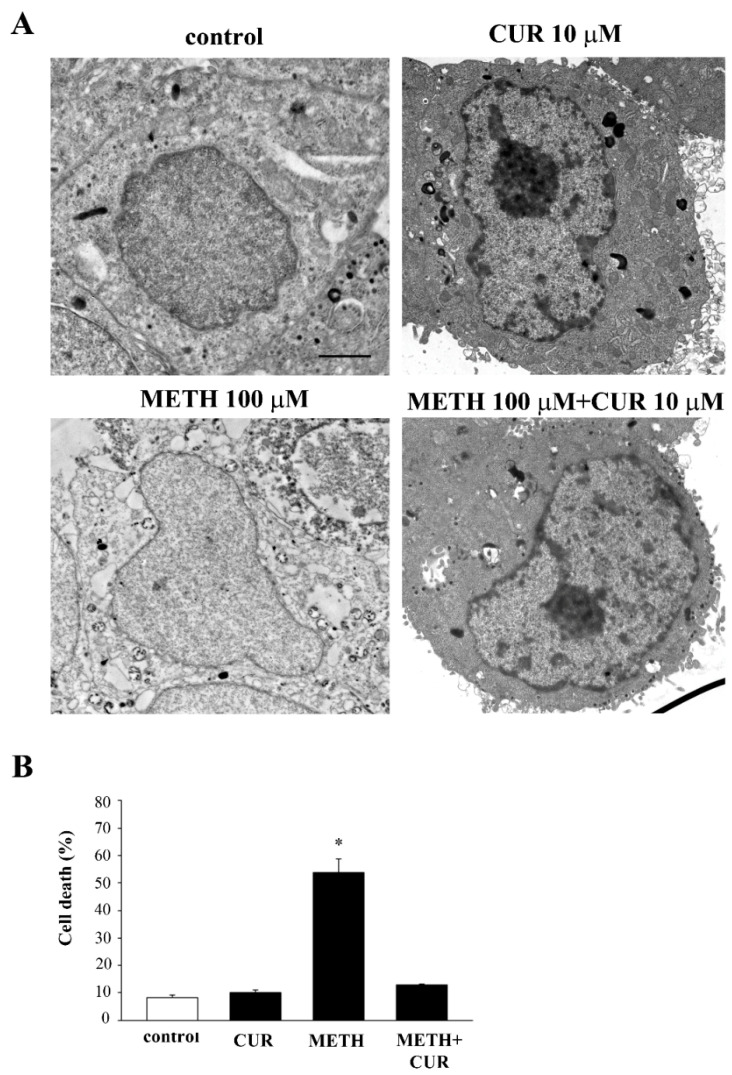
CUR prevents cell death. (**A**) Representative micrographs of PC12 cells showing a typical slow necrosis following METH administration. (**B**) The histogram reports the percentage of cell death (slow necrosis) counted at TEM. * *p* ≤ 0.05 compared with the other groups. Scale bar = 0.5 μM.

It is remarkable that METH per se does not induce significant apoptosis, but induced slow necrosis, as shown by TEM (Figure 19). The full loss of autophagy in the cell makes it impossible to produce the slow necrosis, which instead occurs following METH alone as a result of non-effective autophagy. The absence of any autophagy response generates 3MA-induced apoptosis.

**Figure 20:**
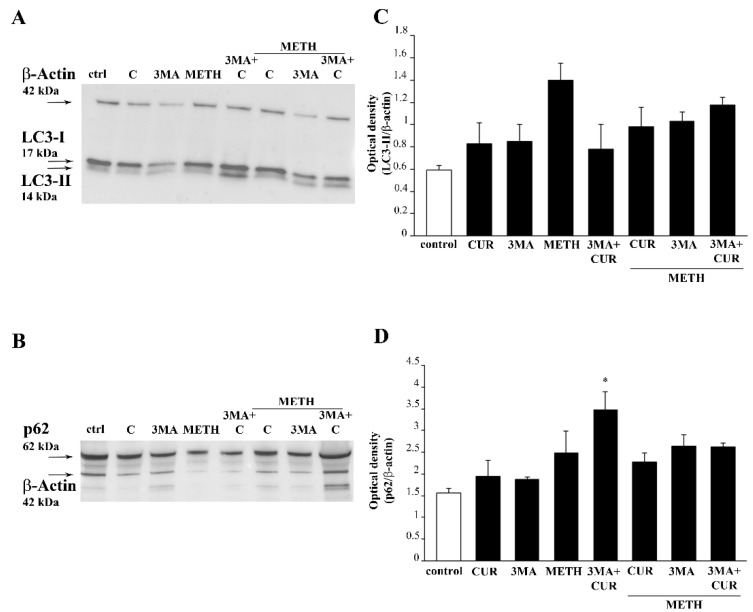
CUR induces autophagy in METH-treated cells. Immunoblotting for LC3-I and LC3-II (**A**) and p62 (**B**) and the graphs reporting the related optical densities (**C** and **D**, respectively). * *p* ≤ 0.05 compared with control.

To strengthen the involvement of autophagy in CUR-induced neuroprotection, the expression of the autophagy protein LC3-II along with the expression of the autophagy protein p62 were measured in the presence of the autophagy inhibitor 3MA. The protein p62 binds ubiquitinated proteins tagged for autophagy degradation, it shuttles substrates within the autophagosome, and it is preferentially degraded by the autophagy machinery [41]. Thus, in the presence of 3MA, which impedes autophagosome formation, a reduction of LC3-II is expected along with accumulation of p62, which cannot be degraded by impaired autophagy. Accordingly, WB assay shows that in METH- treated cells accumulation of both LC3-II and p62 occur. In fact, increased levels of LC3-II induced by METH may represent an early, compensatory response in the attempt to activate autophagy. However, as demonstrated here (Figure 17), METH removes these LC3 particles from the autophagy vacuoles, thus de-potentiating autophagy. Conversely, administration of CUR to METH-treated cells slightly decreases LC3-II and p62 levels compared with METH alone and in combination with 3MA. This is in line with CUR promoting autophagy. These effects of CUR are no longer evident in the presence of 3-MA (Figure 20).

**Figure 21:**
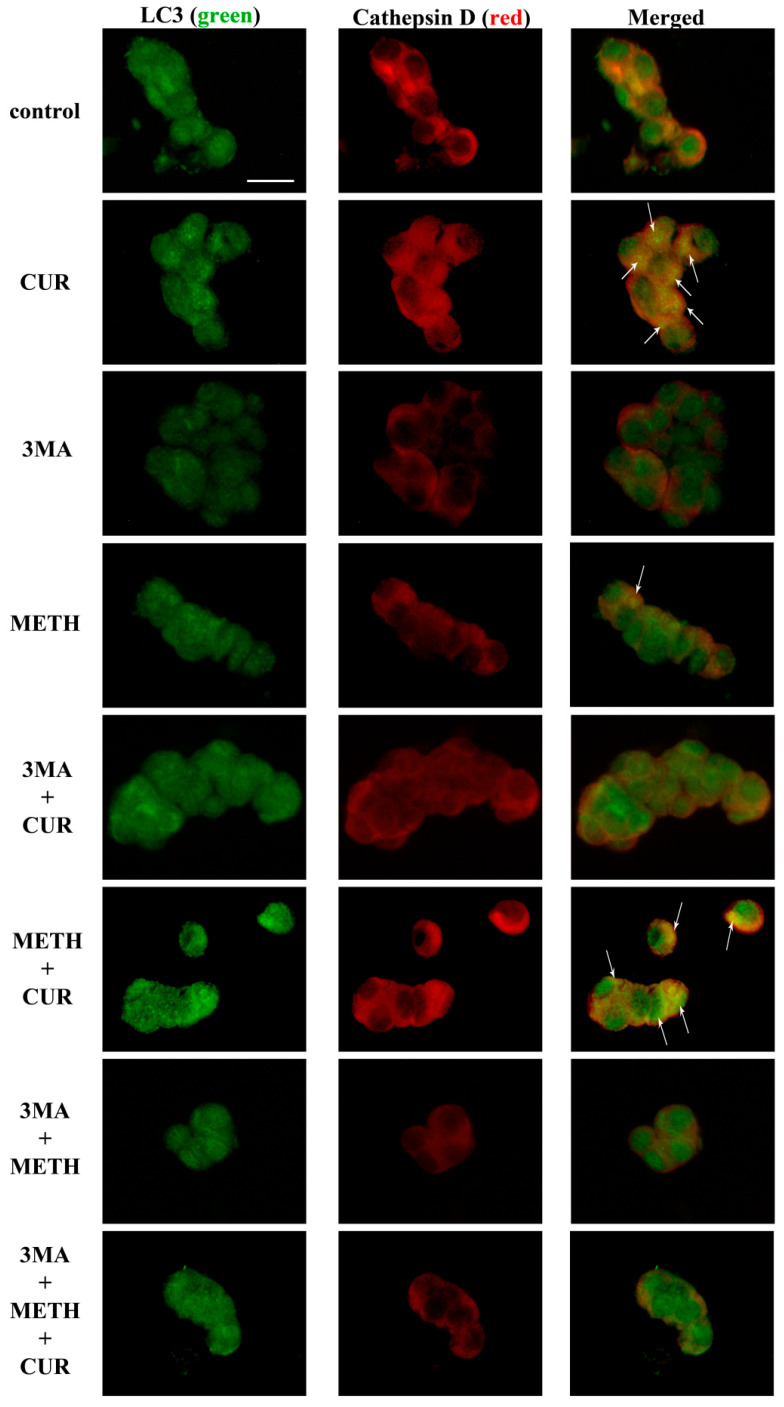
CUR increases, while 3MA reduces, LC3 and cathepsin D co-localization. Representative pictures showing double immunofluorescence for LC3 (green) and cathepsin D (red), and their merge. PC12 cells were treated for 72 h with CUR 10 μM, METH 100 μM, and the autophagy inhibitor 3MA. Arrows indicate cells with intensely merged fluorescence resulting in yellow puncta. Scale bar = 11.4 μM.

These results are substantiated by representative double immunofluorescence for LC3 and a lysosomal marker, cathepsin D. Intensely yellow puncta observed in merged images appear only when CUR is administered to the cell culture, alone or in combination with METH (Figure 21).

This confirms that CUR exerts a pro-autophagic effect in METH-intoxicated cells. In contrast, treatment with 3MA fully prevents merging effect (Figure 21). In the light of these results, we can argue that though CUR slightly induces apoptosis, it does not increase the apoptotic cell death when administered in METH-treated cells. In contrast, CUR treatment, alone or in combination with METH, induces autophagy, which is likely to be responsible for protection against METH-induced toxicity.

## 3. Discussion

In the present manuscript, we describe a powerful protection induced by CUR in METH-intoxicated catecholamine cells. This effect is already described for powerful autophagy inducers such as rapamycin [32]. Since CUR is less powerful and much safer compared with rapamycin as autophagy activator, these data provide a novel scenario to develop and apply this natural molecule to disorders characterized by a deficit in the autophagy machinery. In fact, the molecular mechanisms of CUR-induced neuroprotection are grounded on autophagy induction just counteracting the deleterious influence of METH on the autophagy machinery. This is indicated here by the measurement of the autophagy flux as well as the amount and merging of autophagy and lysosomal proteins. The intimate evidence for opposite modulation of autophagy is provided here by compartmentalization measured at ultrastructural morphometry. However, the strongest evidence is provided by TEM which remains the gold standard to measure the autophagy status. This was implemented by the ultrastructural count of LC3 re-allocation produced by CUR which fixes the dispersion of LC3 out of autophagy vacuoles. The readout and functional significance of such an empowering of the autophagy machinery by CUR is provided here by the CUR-induced clearance of METH-induced α-synuclein accumulation. This effect is likely to be enough in order to produce CUR-induced neuroprotection against METH toxicity. In fact, in METH intoxicated cells a number of misfolded proteins are generated which overwhelms the autophagy pathway. These proteins are expected to produce deleterious effects for cell viability when they are not promptly removed. The occurrence of such a bulk of misfolded proteins in the course of METH intoxication is related to the massive amount of reactive catecholamine metabolites by METH, mostly within DA storage vesicles. α-Synuclein is a sort of gold standard to document such an effect, since as shown elegantly by Conway et al. [21] DA may bind α-synuclein in a way which fosters its oligomerization. This chemical conformation is the one which is supposed to be toxic for the cell. In keeping with this, the present study shows that CUR protects against METH intoxication concomitantly with suppressing α-synuclein accumulation, polymerization likely due to autophagy activation. The ability of CUR to produce neuroprotection may also rely on additional properties of the compound such as the powerful anti-oxidant activity and free radical scavenging properties [42,43,44,45,46,47,48]. However, the autophagy activation per se seems to provide full protection, thus leaving addition mechanisms non-necessary.

Concerning mitochondrial toxicity, it is difficult to discuss this point separately from autophagy induction and such an issue was fully addressed by a recent paper by Lazzeri et al. [32]. In fact, it is well-known that, when autophagy is induced, a simultaneous mitochondrial removal (mitophagy) and mitochondriogenesis occurs. [32,49,50]. In light of these recent findings, and the results provided here, it would be worthwhile to probe the effects of CUR as an epigenetic modulator by aiming specifically those genes which are responsible for autophagy, mitophagy and mitochondriogenesis. Analogous epigenetic investigations are required following CUR administration to see which genes are activated in relationship with the autophagy pathway. Such a CUR-induced plasticity on specific macromolecules and organelles in the cell shed a brand new avenue in drug development.

Thus, even considering that we cannot conclude which specific mechanism of action, if any by itself, is entirely responsible for CUR-induced protection against METH intoxication, the protection achieved was comparable to that described following the gold standard autophagy activator rapamycin.

It is worth of noting that besides a pro-survival effect, the clearance from misfolded α-synuclein and in general the autophagy induction, CUR exerts a phenotypic switch in the cell. In detail, CUR increases the size of PC12 cells. Such an effect is not surprising when considering that we are dealing with a cell line, which derives from an adrenal medullary tumor (i.e., pheochromocytoma). In fact, in previous investigations we found that autophagy induction by an mTOR inhibitor in different strains of tumoral cells, besides producing cell differentiation, also augments cell size [51]. Other natural compounds may be required in order to reproduce the whole range of beneficial effects exerted by the mTOR inhibitor rapamycin. Therefore, we are evaluating other polyphenols which might also produce a strong phenotypic maturation of PC12 cells.

In fact, rapamycin by blocking the mTORC1 complex exerts a number of downstream activities, which may not be shared by CUR. Again, when reviewing data on the effects produced by CUR in relation to the autophagy machinery, we found that the specific effects induced by CUR differ from that induced by rapamycin. In fact, CUR induces autophagy by acting at several molecular levels [52,53,54,55,56]. Again, CUR acts as mTOR inhibitor, which leads to autophagy induction either through activation of ULK1/Atg13 or transcription factor EB (TFEB) [57]. In particular, CUR activates TFEB to promote its translocation to the nucleus, and the subsequent induction of autophagy-related genes [5,6,57,58]. Independently from mTOR upstream molecules, disruption of the mTOR–raptor interaction is another proposed mechanism of curcumin-mediated autophagy activation [59].

It is worth of noting that, despite both METH and CUR produce an increase in LC3 immunofluorescence, the pattern of such a fluorescence is distinct between the two compounds. In detail, while METH produces large spots of LC3, CUR administration produces small, numerous homogeneously placed fluorescent spots. Here we demonstrate that such a different pattern of immunofluorescence depends on the specific pattern of LC3 compartmentalization. In fact, following METH administration LC3 moves from autophagy vacuoles into bigger cytosolic regions compared with the vacuole itself. Contrari wise, the spots produced by CUR are compatible with a small size just like the one own by autophagy vacuoles. In this way immuno-gold stoichiometric molecule detection within specific cell compartments allows to decipher apparent but not real similarities observed at immunofluorescence.

## 4. Materials and Methods

### 4.1. Experimental Design

As first experimental step, we characterized the dose-response effects of CUR on PC12 cell viability. This allowed to select the safety of CUR doses to challenge METH-induced toxicity. Then, we used four doses of METH ranging from 1μM up to 1000 μM, in order to obtain a range of toxicity from moderate to frankly elevate. In preliminary experiments we carried out a time-dependent curve of the effects produced by CUR and METH alone, in order to select the time window where: (i) CUR induces steady and clearly established effects on cell viability; (ii) fully METH toxicity develops producing a toxic plateau. This time interval corresponds in both cases to 72 h, which is the time we referred in the body of the article.

Within this time window, we planned to investigate whether CUR-induced cytotoxicity is mediated via apoptotic or non-apoptotic mechanisms, through flow cytometry analysis, WB and immunofluorescence for caspase 3 and apoptotic cell count at TEM.

Moreover, we analyzed at light microscopy the phenotypic changes induced by CUR or METH alone, along with the potential protective effects of CUR against METH-induced toxicity. These latter effects were evaluated by (i) analyzing cell viability, (ii) evaluating the effects of CUR on METH-induced increase in α-synuclein levels, (iii) determining the involvement of autophagy as potential protective mechanism induced by CUR against METH toxicity. Similarly, TEM and ultrastructural morphometry were carried out in these experimental conditions.

### 4.2. PC12 Cell Culture

The rat pheochromocytoma cell line PC12 was obtained from a cell bank (IRCCS San Martino Institute, Genova, Italy) and grown in RPMI 1640 medium (Sigma-Aldrich, St. Louis, MO, USA) supplemented with 10% heat-inactivated horse serum (HS; Sigma-Aldrich), 5% fetal bovine serum (FBS; Sigma-Aldrich), penicillin (50 IU/mL) and streptomycin (50 mg/mL; Sigma-Aldrich). Cells were grown in 75 cm^2^ tissue culture flask and maintained in a humidified atmosphere containing 5% CO_2_ at 37 °C. Cells were in log phase of growth when used for the experiments.

For light microscopy experiments, 5 × 10^4^ PC12 cells were seeded on polylysine cover slips, which were placed in 24-well plates in a final volume of 1 mL/well. For TEM experiments, 10^6^ cells were seeded in culture dishes in a final volume of 5 mL. For western blotting assays, 5 × 10^5^ cells were seeded in six-well plates in a final volume of 2 mL/well.

### 4.3. Cell Treatments

Stock solutions of CUR and METH were prepared in order to carry out cell treatments. A stock solution of CUR (Sigma-Aldrich) 9.5 mM was obtained by dissolving 3.5 mg of CUR powder in 1 mL of DMSO, while a stock solution of METH (Sigma-Aldrich) 10 mM was prepared by dissolving 2.3 mg of METH directly in 1 mL of culture medium. 3-methyladenine (3MA, Sigma-Aldrich) was dissolved in the culture medium to obtain the treatment solution of 10 mM. Bafilomycin A1 (Sigma-Aldrich) was added to the culture medium to obtain the treatment solution of 50 nM.

Final concentrations of CUR and METH used for the experimental treatments were obtained by diluting appropriate aliquots of the stock solutions within the cell culture medium. In single treatment experiments, PC12 cells were exposed to increasing doses of CUR (0.1 μM, 1 μM, 10 μM, and 50 μM) or METH (1 μM, 10 μM, 100 μM, and 1000 μM) for 72 h. In combined experiments, the CUR doses of 0.1 μM, 1 μM, and 10μM were challenged against METH 100 μM and 1000 μM. In these latter experiments, CUR treatment was carried out 2 h before METH. 3MA was added 30 min before CUR or 2 h before METH. Bafilomycin was added to the culture medium 3 h before cell lysis. 

### 4.4. WST-1 Assay

For WST-1 cell viability assay, PC12 cells were seeded at the density of 10^4^ cells/well and placed within 96-well plates in 100 μL of culture medium. At the end of the treatments, cell viability was assessed using the cell proliferation reagent WST-1 (Roche Diagnostics GmbH, Mannheim, Germany) according to the manufacturer’s protocol. Briefly, 10% WST-1 (4-[3-(4-Iodophenyl)-2-(4-nitrophenyl)-2*H*-5-tetrazolio]-1,3-benzene sulfonate) reagent was added to each well and the cells were incubated for 1 h at 37 °C and 5% CO_2_. Cell viability was measured at 450 nm in a microplate reader (BioTek Instruments, Winooski, VT, USA). Data were obtained in three independent experiments and expressed as the mean percentage ± S.E.M. (assuming control as 100% WST-1).

### 4.5. Trypan Blue Staining

For TB staining, PC12 cells were seeded at a density of 10^4^ cells/well and placed within 24-well plates in 1 mL of culture medium 24 h before treatment. At the end of the treatments, PC12 cells were collected and centrifuged at 800 *g* for 5 min, to obtain a cell pellet. The cell pellet was suspended in the culture medium, and 25 μL of the cell suspension were added to a solution of 1% TB (62.5 μL, Sigma-Aldrich) and PBS (37.5 μL), for 10 min, at room temperature (RT). Ten μL of this solution were used to perform cell count using a Bürker glass chamber. Viable and no-viable cells were counted, and data were expressed as the mean percentage ± S.E.M. of TB-positive cells out of the total cells. Data were obtained in three independent experiments.

### 4.6. Fluoro Jade B

PC12 cells were washed in PBS and fixed with 4% paraformaldehyde for 5 min at RT. Fixed cells were first incubated with 0.06% potassium permanganate for 10 min at RT and then washed with distilled water. Then the cells were incubated with 0.0004% FJB (Merck Millipore. Billerica, MA, USA) solution (consisting in 0.01% FJB in acetic acid) at RT for 20 min, cover slipped with mounting medium. FJB-positive cells were analyzed at Nikon Eclipse 80i light microscopy (Nikon, Tokyo, Japan), equipped with a florescence lamp and a digital camera connected to the NIS Elements software for image analysis (Nikon). For each experimental group, the count of FJB-positive cells and the measure of the fluorescence intensity were carried out. In detail, the number of FJB-positive cells was counted at 20× magnification within five distinct microscopic fields, where only distinct, not overlapped cells were counted. The intensity of the fluorescent signaling was measured under florescence microscopy in 50 cells/group, using the software Image J (company, city, state abbrev if USA, country). Values were expressed as the mean number ± S.E.M. of FJB-positive cells and the mean percentage ± S.E.M. of optical density for each experimental group. Data refer to three independent experiments.

### 4.7. CytofluorimetricAanalysis of Subdiploid (subG1) DNA Peak

Apoptosis was assessed by DNA fluorescence flow cytometric profiles following the method described by Nicoletti et al. [60]. Briefly, cells detached from plates were washed two-fold in PBS, then resuspended in 1 mL of fluorochromic solution containing 0.05 mg/mL PI (propidium iodide), 0.1% sodium citrate and 0.1% Triton X-100 and then placed at 4 °C in the dark overnight before the flow-cytometric analysis. The fluorescence of DNA of isolated nuclei (PI fluorescence) was analyzed by Cytoflexflow cytometer (Beckman Coulter, Cassina de’Pecchi, Milano, Italy) and the percentage of apoptotic nuclei (subdiploid DNA peak in the DNA fluorescence histogram) was calculated using CytExpert v.2.3 software (company, city, state abbrev if USA, country).

### 4.8. Haematoxylin and Eosin Histochemistry

Cells were fixed with 4% paraformaldehyde in PBS for 15 min at RT, washed with PBS and then stained with haematoxylin solution (Sigma-Aldrich). Haematoxylin staining was stopped by washing in running water. Then the cells were stained with eosin solution (Sigma-Aldrich). After repeated washing with distilled water to remove the excess of dye, cells were dehydrated in increasing alcohol solutions, clarified in xylene, and finally covered with DPX mounting medium (Sigma-Aldrich). H&E stained cells were observed under Nikon Eclipse 80i light microscope (Nikon). In order to evaluate cell viability, H&E-stained cells were counted under 20× magnification. Cell counts were carried out within 5 distinct microscopic fields, where only distinct, not overlapped cells were counted. Values are expressed as the mean percentage ± S.E.M. of cells counted in three independent experiments (assuming control cells as 100%).

H&E-stained cells were used also for determining phenotypic changes induced by the treatments. In particular, the maximum cell diameter and the ratio between maximum and minimum cell diameter, here indicated as polarization index, were measured. Cells were analyzed at light microscopy, at 40× magnification, and measures were carried out through the software Image J. For this purpose, 50 cells/group were measured. Values related to the maximum cell diameter were expressed as the mean ± S.E.M. of the maximum diameter measured in each experimental group. Polarization index was expressed as the mean value ± S.E.M. of the ratio between maximum and minimum cell diameter measured in each group. Data refer to three independent experiments.

### 4.9. Immunocytochemistry at Light Microscopy

For immunocytochemistry PC12 cells were washed in PBS, fixed with 4% paraformaldehyde for 5 min at RT and permeabilized by Triton X 0.1% (Sigma-Aldrich) for 15 min in PBS.

For α-synuclein immune-peroxidase experiments, cells were incubated in 3% hydrogen peroxide (H_2_O_2_) for 20 min at RT to block endogenous peroxidase activity, followed by a blocking solution containing 10% normal goat serum (NGS) in PBS for 1 h at RT. Then they were incubated overnight at 4°C with the primary antibody solution containing the anti-*α*-synuclein primary antibody (Abcam, Cambridge, UK) diluted 1:1000 and 2% NGS in PBS. The antigen-antibody reaction was revealed using the anti-mouse biotin-conjugated secondary antibody (Vector Laboratories, Burlingame, CA, USA) diluted 1:200 for 1 h at RT, followed by avidin-biotin complex (ABC, Vector) for 1 h and the peroxidase substrate diaminobenzidine (DAB, Vector) for 3 min at RT. Finally, cells were dehydrated in increasing alcohol solutions. All these reactions were carried out within the well plate. After washing in PBS and clarified in xylene, slices were gently pulled out, cover-slipped with DPX mounting medium (Sigma-Aldrich) and observed using light microscopy (Nikon).

For immunofluorescence, cells were incubated overnight at 4 °C with the primary antibody solution containing the anti-LC3 antibody (Abcam) diluted 1:75, anti-caspase 3 antibody (Cell Signaling Technology, Danvers, MA, USA) diluted 1:100, or anti-cathepsin D (Sigma-Aldrich) diluted 1:1000 in 2% NGS and PBS. Afterwards, cells were incubated for 1 h with the anti-rabbit fluorophore-conjugated secondary antibodies (Alexa 488, Life Technologies, Carlsbad, CA, USA) diluted 1:200 or anti-mouse fluorophore-conjugated secondary antibodies (Alexa 546; Life Technologies) diluted 1:200 in PBS at RT. All these reactions were carried out within the well plate. After washing in PBS, slices were gently pulled out, transferred on a coverslip and mounted with the mounting medium Fluoroshield (Sigma-Aldrich). Cells were observed under fluorescence microscopy (Nikon).

Densitometric analysis of LC3-immunofluorescent cells were carried out under fluorescent microscopy using the software IMAGEJ. Values related to 50 cells/group were re as the mean ± S.E.M. of the optical density obtained in each group. All data represent the mean from three independent experiments.

### 4.10. Western Blotting

PC12 cells were lysed in a buffer (100 mM Tris-HCl, pH 7.5, 5 M NaCl, 0.5 m EDTA, 10% SDS, 1% NP40, IGEPAL), containing protease and phosphatase inhibitors, and centrifuged at 15,000× *g* for 20 min at 4 °C. The supernatant was collected, and protein concentration was determined using a protein assay kit (Sigma-Aldrich). Samples containing 40 μg of total proteins were solubilized and electrophoresed on a 12% sodium dodecyl sulphate- (SDS-) polyacrylamide gel. Following electrophoresis, proteins were transferred to the nitrocellulose membrane (Bio-Rad Laboratories, Milano, Italy). The membrane was immersed in a blocking solution (3% nonfat dried milk in 20 mM Tris and 137 mM NaCl at pH 7.6 containing 0.05% Tween-20) for 2 h on a plate shaker. Subsequently, the membrane was incubatedovernight at 4 °C on the plate shaker with the following primary antibodies: mouse anti-α-synuclein (1:800; Abcam), rabbit anti-caspase 3 (1:1000; Cell Signaling Technology, Danvers, MA, USA), rabbit anti-LC3-I and LC3-II (1:1000; Cell Signaling Technology), and rabbit anti-p62 (1:1000; Sigma-Aldrich). Blot was probed with horseradish peroxidase-labeled secondary antibody, and the bands were visualized with enhanced chemiluminescence reagents (Bio-Rad Laboratories). Image analysis was carried out by ChemiDoc System (Bio-Rad Laboratories).

The intensity of the blotting was measured using the software Image J and expressed as the mean ± S.E.M. of the optical density for each experimental group, calculated in three independent experiments.

In experiments with bafilomycin A1, cells were lysed and proteins electrophoretically resolved as previously reported [61]. Then, separated proteins were electro-transferred onto nitrocellulose membranes (Schleicher & Schuell, Maidstone, UK) by a semi-dry system (Novablot, Pharmacia Biotech, Cologno Monzese, Milano, Italy). Membranes were blocked with 3% non-fat milk in PBS and then incubated (overnight at 4 °C) with anti-LC3-I and LC3-II (Sigma L7543). After extensive washing with PBS containing 0.1% Tween-20 (TBST), blots were incubated with 1:10,000 dilution of HRP-conjugated secondary antibody (Bio-Rad Laboratories) for 1 h at room temperature. Immunopositive bands were detected with a chemiluminescence detection system (GE Healthcare Biosciences, Piscataway, NJ, USA). To check for equal loading of the gel, membranes were probed with mouse anti-β-actin (1:5000, A5441, Sigma). Densitometric analysis was performed with the Quantity One software (Bio-Rad Laboratories).

### 4.11. Transmission Electron Microscopy

PC12 cells were centrifuged at 1000 *g* for 5 min and the supernatant was removed. Cell pellet was fixed in 2.0% paraformaldehyde and 0.1% glutaraldehyde in 0.1 M PBS, pH 7.4 for 90 min at 4 °C. This fixing solution contains a concentration of aldehyde which minimally covers antigen epitopes, while fairly preserving tissue architecture. After washing, specimens were post-fixed in 1% OsO_4_ for 1 h at 4 °C; they were dehydrated in ethanol and finally embedded in epoxy resin.

Apoptotic cells were identified in plain TEM through typical apoptotic features such as apoptotic nuclear bodies with condensed chromatin and disarranged cytoplasm.

Plain TEM was implemented by a post-embedding immunocytochemistry procedure for antibodies against LC3 used as markers of autophagy accordingly to the manuscript “Guidelines for the Use and Interpretation of Assays for Monitoring Autophagy (4th Edition)” [62].

At the end of plain TEM or immunocytochemistry procedure, ultrathin sections were stained with uranyl acetate and lead citrate, and they were finally examined using a JEOL JEM-100SX transmission electron microscope (JEOL, Tokyo, Japan). For ultrastructural morphometryand apoptotic cell count, grids containing non serial ultrathin sections (40–50 nm thick) were examined at TEM, at a magnification of 8000×. Several grids were analyzed in order to count a total number of 50 cells for each experimental group.

### 4.12. Postembedding Immunocytochemistry

In our previous studies we validated the combination of aldehydes, OsO_4_, and epoxy resin for immuno-gold-based ultrastructural morphometry [63]. In fact, our protocol allows a minimal epitope covering, while preserving cell ultrastructure [63,64,65]. In particular, OsO_4_, binding to cell membrane, enhances the contrast of cytosolic organelles, and it prevents the formation of membrane’s artifacts, which may mimic vacuoles. Moreover, epoxy resin preserves cell morphology respect to the classic acrylic resin used for immunocytochemistry. Post-embedding procedure was carried out on ultrathin sections collected on nickel grids. The grids were incubated on droplets of aqueous sodium meta-periodate (NaIO_4_), for 30 min, at room temperature in order to remove OsO_4_ and allowing a closer contact between antibodies and antigens [64].

This step allows a better visualization of immuno-gold particles specifically located within a sharp context of cell integrity, in order to counting molecules within specific cell compartments. After washing in PBS, the grids were incubated in a blocking solution containing 10% goat serum and 0.2% saponin for 20 min, at room temperature. Grids were then incubated with the primary antibody solution containing rabbit anti-LC3 (Abcam, diluted 1:50) with 0.2% saponin and 1% goat serum in a humidified chamber overnight, at 4 °C. After washing in PBS, grids were incubated with the secondary antibodies conjugated with gold particles (20 nm mean diameter, for gold particle anti-rabbit, BB International), diluted 1:30 in PBS containing 0.2% saponin and 1% goat serum for 1 h, at RT. Control sections were incubated with the secondary antibody only.

### 4.13. Ultrastructural Morphometry

For ultrastructural morphometry, grids containing non serial ultrathin sections (40–50 nm thick) were examined at TEM, at a magnification of 8000×. Several grids were analyzed in order to count a total number of 50 cells for each experimental group. We counted the number of LC3-positive vacuoles per cell as vacuoles with single, double, or multiple membranes possessing the same electron density of the surrounding cytoplasm or partly containing some electron dense structure.

### 4.14. Statistical Analysis

For cell viability experiments, WST-1 activity was measured at 450 nm as optical density and expressed as the mean percentage ± S.E.M. (assuming control as 100% WST-1) calculated in three independent experiments. Values obtained in TB experiments were expressed as percentage of TB-positive cells ± S.E.M. counted in three independent experiments. The number of FJB-positive cells was counted at 20× magnification within five distinct microscopic fields and expressed as the mean number ± S.E.M. of FJB-positive cells. The intensity of the fluorescent signaling was measured at 40× magnification in 50 cells/group, using the software IMAGE J. Values are expressed as the mean percentage ± S.E.M. of the optical density. All data refer to three independent experiments.

For determining phenotypic changes induced by different treatments, the maximum cell diameter and the ratio between maximum and minimum cell diameter, here indicated as Polarization Index, were measured. H&E stained cells were analyzed at light microscopy, at 40× magnification, and measures were carried out through the software IMAGE J. For this purpose, 50 cells/group were measured. Values related to the maximum cell diameter were expressed as the mean ± S.E.M. of the maximum diameter measured in each experimental group. Polarization Index was expressed as the mean value ± S.E.M. of the ratio between maximum and minimum cell diameter measured in each group. All data refer to three independent experiments.

For LC3 immunofluorescence, densitometric analysis was carried out using the software IMAGEJ at 40× magnification. Data were obtained from 50 cells/group and they are expressed as the mean ± S.E.M. of the optical density obtained in three independent experiments.

For western blot assay, the intensity of the blotting was measured through Image J and expressed as the mean ± S.E.M. of the optical density for each experimental group, calculated in three independent experiments.

Inferential statistics to compare groups was carried out by using One-way analysis of variance, ANOVA, followed by Scheffè’s post-hoc analysis. Differences between groups were considered statistically significant when the null hypothesis (H0) was *p* ≤ 0.05.

## Data Availability

The data used to support the findings of this study are available from the corresponding author upon request.

## Supplementary Materials

Supplementary Figures are available online, Figure S1: (Time-dependency of the effects of increasing doses of CUR on cell viability); Figure S2: (Time-dependency of the effects of increasing doses of METH on cell viability); Figure S3: (Inhibition of autophagy in METH-treated cells induces cell death); Figure S4: (Autophagy inhibition increases Fluoro-Jade B-responsiveness of PC12 cells after METH); Figure S5: (Autophagy inhibition increases morphological alterations of PC12 cells after METH).

## References

[1] Aggarwal B.B., Harikumar K.B. (2009). Potential therapeutic effects of curcumin, the anti-inflammatory agent, against neurodegenerative, cardiovascular, pulmonary, metabolic, autoimmune and neoplastic diseases. Int. J. Biochem. Cell Biol..

[2] Lim G.P., Chu T., Yang F., Beech W., Frautschy S.A., Cole G.M. (2001). The curry spice curcumin reduces oxidative damage and amyloid pathology in an Alzheimer transgenic mouse. J. Neurosci..

[3] Menon V.P., Sudheer A.R. (2007). Antioxidant and anti-inflammatory properties of curcumin. Adv. Exp. Med. Biol..

[4] Wang W., Xu J. (2020). Curcumin Attenuates Cerebral Ischemia-reperfusion Injury Through Regulating Mitophagy and Preserving Mitochondrial Function. Curr. Neurovasc. Res..

[5] Song J.X., Sun Y.R., Peluso I., Zeng Y., Yu X., Lu J.H., Xu Z., Wang M.Z., Liu L.F., Huang Y.Y. (2016). A novel curcumin analog binds to and activates TFEB in vitro and in vivo independent of MTOR inhibition. Autophagy.

[6] Zhang J., Wang J., Xu J., Lu Y., Jiang J., Wang L., Shen H.M., Xia D. (2016). Curcumin targets the TFEB-lysosome pathway for induction of autophagy. Oncotarget.

[7] He X.J., Uchida K., Megumi C., Tsuge N., Nakayama H. (2015). Dietary curcumin supplementation attenuates 1-methyl-4-phenyl-1,2,3,6-tetrahydropyridine (MPTP) neurotoxicity in C57BL mice. J. Toxicol. Pathol..

[8] Xia X.J., Lian Y.G., Zhao H.Y., Xu Q.L. (2016). Curcumin protects from oxidative stress and inhibits α-synuclein aggregation in MPTP induced parkinsonian mice. Int. J. Clin. Exp. Med..

[9] Wang J., Du X.X., Jiang H., Xie J.X. (2009). Curcumin attenuates 6-hydroxydopamine-induced cytotoxicity by anti-oxidation and nuclear factor-kappa B modulation in MES23.5 cells. Biochem. Pharmacol..

[10] Jaisin Y., Thampithak A., Meesarapee B., Ratanachamnong P., Suksamrarn A., Phivthong-Ngam L., Phumala-Morales N., Chongthammakun S., Govitrapong P., Sanvarinda Y. (2011). Curcumin I protects the dopaminergic cell lines SH-SY5Y from 6-hydroxydopamine-induced neurotoxicity through attenuation of p53-mediated apoptosis. Neurosci. Lett..

[11] Maiti P., Dunbar G.L. (2018). Use of Curcumin, a Natural Polyphenol for Targeting Molecular Pathways in Treating Age-Related Neurodegenerative Diseases. Int. J. Mol. Sci..

[12] Callaghan R.C., Cunningham J.K., Sykes J., Kish S.J. (2012). Increased risk of Parkinson’s disease in individuals hospitalized with conditions related to the use of methamphetamine or other amphetamine-type drugs. Drug Alcohol Depend..

[13] Ares-Santos S., Granado N., Espadas I., Martinez-Murillo R., Moratalla R. (2014). Methamphetamine causes degeneration of dopamine cell bodies and terminals of the nigrostriatal pathway evidenced by silver staining. Neuropsychopharmacology.

[14] Curtin K., Fleckenstein A.E., Robison R.J., Crookston M.J., Smith K.R., Hanson G.R. (2015). Methamphetamine/amphetamine abuse and risk of Parkinson’s disease in Utah: A population-based assessment. Drug Alcohol Depend..

[15] Weinshenker D. (2018). Long Road to Ruin: Noradrenergic Dysfunction in Neurodegenerative Disease. Trends Neurosci..

[16] Fornai F., Puglisi-Allegra S. (2021). Autophagy status as a gateway for stress-induced catecholamine interplay in neurodegeneration. Neurosci. Biobehav. Rev..

[17] Fornai F., Giorgi F.S., Bassi L., Ferrucci M., Alessandrì M.G., Corsini G.U. (2000). Modulation of dihydroxyphenylacetaldehyde extracellular levels in vivo in the rat striatum after different kinds of pharmacological treatment. Brain Res..

[18] Gesi M., Santinami A., Ruffoli R., Conti G., Fornai F. (2001). Novel aspects of dopamine oxidative metabolism (confounding outcomes take place of certainties). Pharmacol. Toxicol..

[19] LaVoie M.J., Hastings T.G. (1999). Dopamine quinone formation and protein modification associated with the striatal neurotoxicity of methamphetamine: Evidence against a role for extracellular dopamine. J. Neurosci..

[20] Hastings T.G., Lewis D.A., Zigmond M.J. (1996). Role of oxidation in the neurotoxic effects of intrastriatal dopamine injections. Proc. Natl. Acad. Sci. USA.

[21] Conway K.A., Rochet J.-C., Bieganski R.M., Lansbury P.T. (2001). Kinetic stabilization of the α-synuclein protofibril by a dopamine-α-synuclein adduct. Science.

[22] Sulzer D. (2001). alpha-synuclein and cytosolic dopamine: Stabilizing a bad situation. Nat. Med..

[23] Fornai F., Lenzi P., Gesi M., Soldani P., Ferrucci M., Lazzeri G., Capobianco L., Battaglia G., De Blasi A., Nicoletti F. (2004). Methamphetamineproducesneuronalinclusions in the nigrostriatalsystem and in PC12 cells. J. Neurochem..

[24] Quan L., Ishikawa T., Michiue T., Li D.R., Zhao D., Oritani S., Zhu B.L., Maeda H. (2005). Ubiquitin-immunoreactive structures in the midbrain of methamphetamine abusers. Leg Med..

[25] Fornai F., Soldani P., Lazzeri G., di Poggio A.B., Biagioni F., Fulceri F., Batini S., Ruggieri S., Paparelli A. (2005). Neuronalinclusions in degenerative disorders. Do they represent static features or a key to understand the dymanics of the disease?. Brain Res. Bull..

[26] Schmued L.C., Hopkins K.J. (2000). Fluoro-Jade B: A high affinity fluorescent marker for the localization of neuronal degeneration. Brain Res..

[27] Schmuck G., Kahl R. (2009). The use of Fluoro-Jade in primary neuronal cell cultures. Arch. Toxicol..

[28] Ullah I., Ullah N., Naseer M.I., Lee H.Y., Kim M.O. (2012). Neuroprotection with metformin and thymoquinone against ethanol-induced apoptotic neurodegeneration in prenatal rat cortical neurons. BMC Neurosci..

[29] Asanuma M., Miyazaki I., Higashi Y., Diaz-Corrales F.J., Shimizu M., Miyoshi K., Ogawa N. (2007). Suppression of p53-activated gene, PAG608, attenuates methamphetamine-induced neurotoxicity. Neurosci. Lett..

[30] Castino R., Lazzeri G., Lenzi P., Bellio N., Follo C., Ferrucci M., Fornai F., Isidoro C. (2008). Suppression of autophagy precipitates neuronal cell death following low doses of methamphetamine. J. Neurochem..

[31] Wu J., Zhu D., Zhang J., Li G., Liu Z., Sun J. (2015). Lithium protects against methamphetamine-induced neurotoxicity in PC12 cells via Akt/GSK3β/mTOR pathway. Biochem. Biophys. Res. Commun..

[32] Lazzeri G., Biagioni F., Fulceri F., Busceti C.L., Scavuzzo M.C., Ippolito C., Salvetti A., Lenzi P., Fornai F. (2018). mTOR Modulates Methamphetamine-Induced Toxicity through Cell Clearing Systems. Oxid. Med. Cell Longev..

[33] Cubells J.F., Rayport S., Rajendran G., Sulzer D. (1994). Methamphetamine neurotoxicity involves vacuolation of endocytic organelles and dopamine-dependent intracellular oxidative stress. J. Neurosci..

[34] Lazzeri G., Lenzi P., Busceti C.L., Ferrucci M., Falleni A., Bruno V., Paparelli A., Fornai F. (2007). Mechanisms involved in the formation of dopamine-induced intracellular bodies within striatal neurons. J. Neurochem..

[35] Fornai F., Lenzi P., Lazzeri G., Ferrucci M., Fulceri F., Giorgi F.S., Falleni A., Ruggieri S., Paparelli A. (2007). Fine ultrastructure and biochemistry of PC12 cells: A comparative approach to understand neurotoxicity. Brain Res..

[36] Yamamoto A., Tagawa Y., Yoshimori T., Moriyama Y., Masaki R., Tashiro Y. (1998). Bafilomycin A1 prevents maturation of autophagic vacuoles byinhibiting fusion between autophagosomes and lysosomes in rat hepatomacell line, H-4-II-E cells. Cell Struct. Funct..

[37] Boya P., González-Polo R.A., Casares N., Perfettini J.L., Dessen P., Larochette N., Métivier D., Meley D., Souquere S., Yoshimori T. (2005). Inhibition of macroautophagy triggers apoptosis. Mol. Cell Biol..

[38] Mizushima N., Yoshimori T. (2007). How to interprete LC3 immunoblotting. Autophagy.

[39] Seglen P.O., Gordon P.B. (1982). 3-Methyladenine: Specific inhibitor of autophagic/lysosomal protein degradation in isolated rat hepatocytes. Proc. Natl. Acad. Sci. USA.

[40] Blommaart E.F., Krause U., Schellens J.P. (1997). The phosphatidylinositol 3-kinase inhibitors wortmannin and LY294002 inhibit autophagy in isolated rat hepatocytes. Eur. J. Biochem..

[41] Bjørkøy G., Lamark T., Pankiv S., Øvervatn A., Brech A., Johansen T. (2009). Monitoring autophagic degradation of p62/SQSTM1. Methods Enzymol..

[42] Kim D.S., Park S.Y., Kim J.K. (2001). Curcuminoids from *Curcuma longa* L. (Zingiberaceae) that protect PC12 rat pheochromocytoma and normal human umbilical vein endothelial cells from betaA(1-42) insult. Neurosci. Lett..

[43] Mendonça L.M., da Silva Machado C., Teixeira C.C., de Freitas L.A., de Lourdes Pires Bianch M., Antunes L.M. (2013). Curcumin reduces cisplatin-induced neurotoxicity in NGF-differentiated PC12 cells. Neurotoxicology.

[44] Chang C.H., Chen H.X., Yü G., Peng C.C., Peng R.Y. (2014). Curcumin-Protected PC12 Cells Against Glutamate-Induced Oxidative Toxicity. Food Technol. Biotechnol..

[45] Rahaman M.S., Banik S., Akter M., Rahman M.M., Sikder M.T., Hosokawa T., Saito T., Kurasaki M. (2020). Curcumin alleviates arsenic-induced toxicity in PC12 cells via modulating autophagy/apoptosis. Ecotoxicol. Environ. Saf..

[46] Siddiqui M.A., Kashyap M.P., Kumar V., Tripathi V.K., Khanna V.K., Yadav S., Pant A.B. (2011). Differential protection of pre-, co- and post-treatment of curcumin against hydrogen peroxide in PC12 cells. Hum. Exp. Toxicol..

[47] Ao G.Z., Chu X.J., Ji Y.Y., Wang J.W. (2014). Antioxidant properties and PC12 cell protective effects of a novel curcumin analogue (2E,6E)-2,6-bis(3,5- dimethoxybenzylidene)cyclohexanone (MCH). Int. J. Mol. Sci..

[48] Chakraborty S., Karmenyan A., Tsai J.W., Chiou A. (2017). Inhibitory effects of curcumin and cyclocurcumin in 1-methyl-4-phenylpyridinium (MPP+) induced neurotoxicity in differentiated PC12 cells. Sci. Rep..

[49] Palikaras K., Tavernarakis N. (2014). Mitochondrial homeostasis: The interplay between mitophagy and mitochondrial biogenesis. Exp. Gerontol..

[50] Ferese R., Lenzi P., Fulceri F., Biagioni F., Fabrizi C., Gambardella S., Familiari P., Frati A., Limanaqi F., Fornai F. (2020). Quantitative Ultrastructural Morphometry and Gene Expression of mTOR-Related Mitochondriogenesis within Glioblastoma Cells. Int. J. Mol. Sci..

[51] Ferrucci M., Biagioni F., Lenzi P., Gambardella S., Ferese R., Calierno M.T., Falleni A., Grimaldi A., Frati A., Esposito V. (2017). Rapamycin promotes differentiation increasing βIII-tubulin, NeuN, and NeuroD while suppressing nest inexpression in glioblastoma cells. Oncotarget.

[52] Shinojima N., Yokoyama T., Kondo Y., Kondo S. (2007). Roles of the Akt/mTOR/p70S6K and ERK1/2 signaling pathways in curcumin-induced autophagy. Autophagy.

[53] Aoki H., Takada Y., Kondo S., Sawaya R., Aggarwal B.B., Kondo Y. (2007). Evidence that curcumin suppresses the growth of malignant gliomas in vitro and in vivo through induction of autophagy: Role of Akt and extracellular signal-regulated kinase signaling pathways. Mol. Pharmacol..

[54] Hasima N., Ozpolat B. (2014). Regulation of autophagy by polyphenolic compounds as a potential therapeutic strategy for cancer. Cell Death Dis..

[55] Su J., Zhou X., Yin X., Wang L., Zhao Z., Hou Y., Zheng N., Xia J., Wang Z. (2017). The effects of curcumin on proliferation, apoptosis, invasion, and NEDD4 expression in pancreatic cancer. Biochem. Pharmacol..

[56] Shakeri A., Cicero A.F.G., Panahi Y., Mohajeri M., Sahebkar A. (2019). Curcumin: A naturally occurring autophagy modulator. J. Cell. Physiol..

[57] Limanaqi F., Biagioni F., Busceti C.L., Ryskalin L., Polzella M., Frati A., Fornai F. (2019). Phytochemicals Bridging Autophagy Induction and Alpha-Synuclein Degradation in Parkinsonism. Int. J. Mol. Sci..

[58] Wu Y., Liang S., Xu B., Zhang R., Xu L. (2018). Protective effect of curcumin on dopamine neurons in Parkinson’s disease and its mechanism. Zhejiang Da Xue Xue Bao Yi Xue Ban.

[59] Beevers C.S., Chen L., Liu L., Luo Y., Webster N.J., Huang S. (2009). Curcumin disrupts the Mammalian target of rapamycin-raptor complex. Cancer Res..

[60] Riccardi C., Nicoletti I. (2006). Analysis of apoptosis by propidium iodide staining and flow cytometry. Nat. Protoc..

[61] Fabrizi C., Pompili E., De Vito S., Somma F., Catizone A., Ricci G., Lenzi P., Fornai F., Fumagalli L. (2016). Impairment of the autophagic flux in astrocytes intoxicated by trimethyltin. Neurotoxicology.

[62] Klionsky D.J., Abdel-Aziz A.K., Abdelfatah S., Abdellatif M., Abdoli A., Abel S., Abeliovich H., Abildgaard M.H., Abudu Y.P., Acevedo-Arozena A. (2021). Guidelines for the use and interpretation of assays for monitoring autophagy (4th edition). Autophagy.

[63] Lenzi P., Lazzeri G., Biagioni F., Busceti C.L., Gambardella S., Salvetti A., Fornai F. (2016). The Autophagoproteasome—A Novel Cell Clearing Organelle in Baseline and Stimulated Conditions. Front. Neuroanat..

[64] Bendayan M., Zollinger M. (1983). Ultrastructural localization of antigenic sites on osmium-fixed tissues applying the protein A-gold technique. J. Histochem. Cytochem..

[65] D’Alessandro D., Mattii L., Moscato S., Bernardini N., Segnani C., Dolfi A., Bianchi F. (2004). Immunohistochemical demonstration of the small GTPaseRhoAA on epoxy-resin embedded sections. Micron.

