# Supplementary material for: Neuroprotective Effects of Curcumin in Methamphetamine-Induced Toxicity
Source: Molecules. 2021 Apr 24;26(9):2493. doi: 10.3390/molecules26092493 (PMC8123176; doi:10.3390/molecules26092493)
Supplement: Supplementary file 1 [file molecules-26-02493-s001.zip › molecules-1166464-supplementary.pdf]

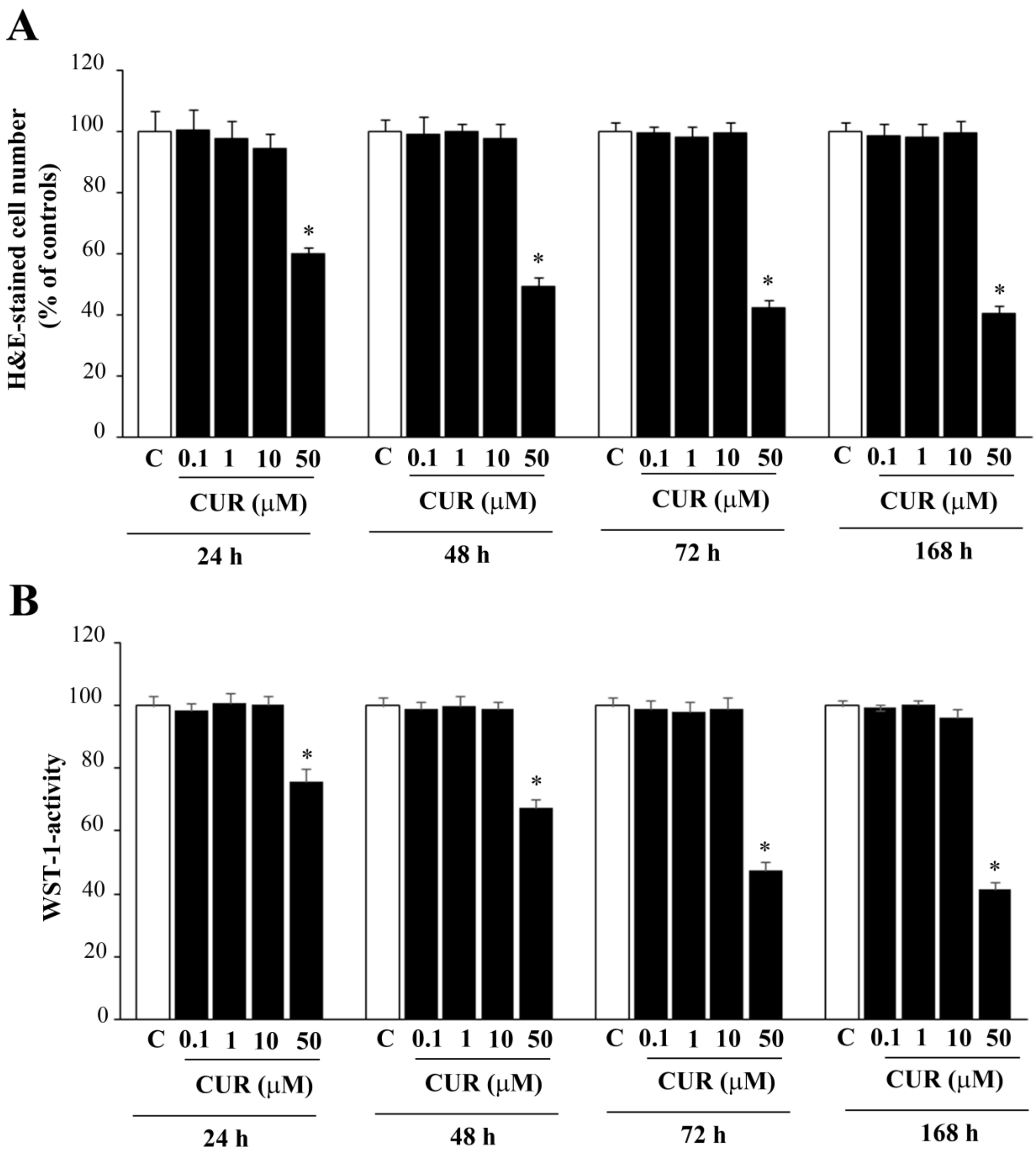

Supplementary Figure 1. Time-dependency of the effects of increasing doses of CUR on cell viability. PC12 cell viability after increasing times (from 24 hours up to 168 hours) and doses (from 0.1 μM up to 50 μM) of CUR, assessed by H&E and WST-1 assay, is reported in the graphs shown in (**A**) and (**B**), respectively. Only the highest dose of CUR (50 μM) affects cell viability at all times challenged. *p<0.05 compared with control.

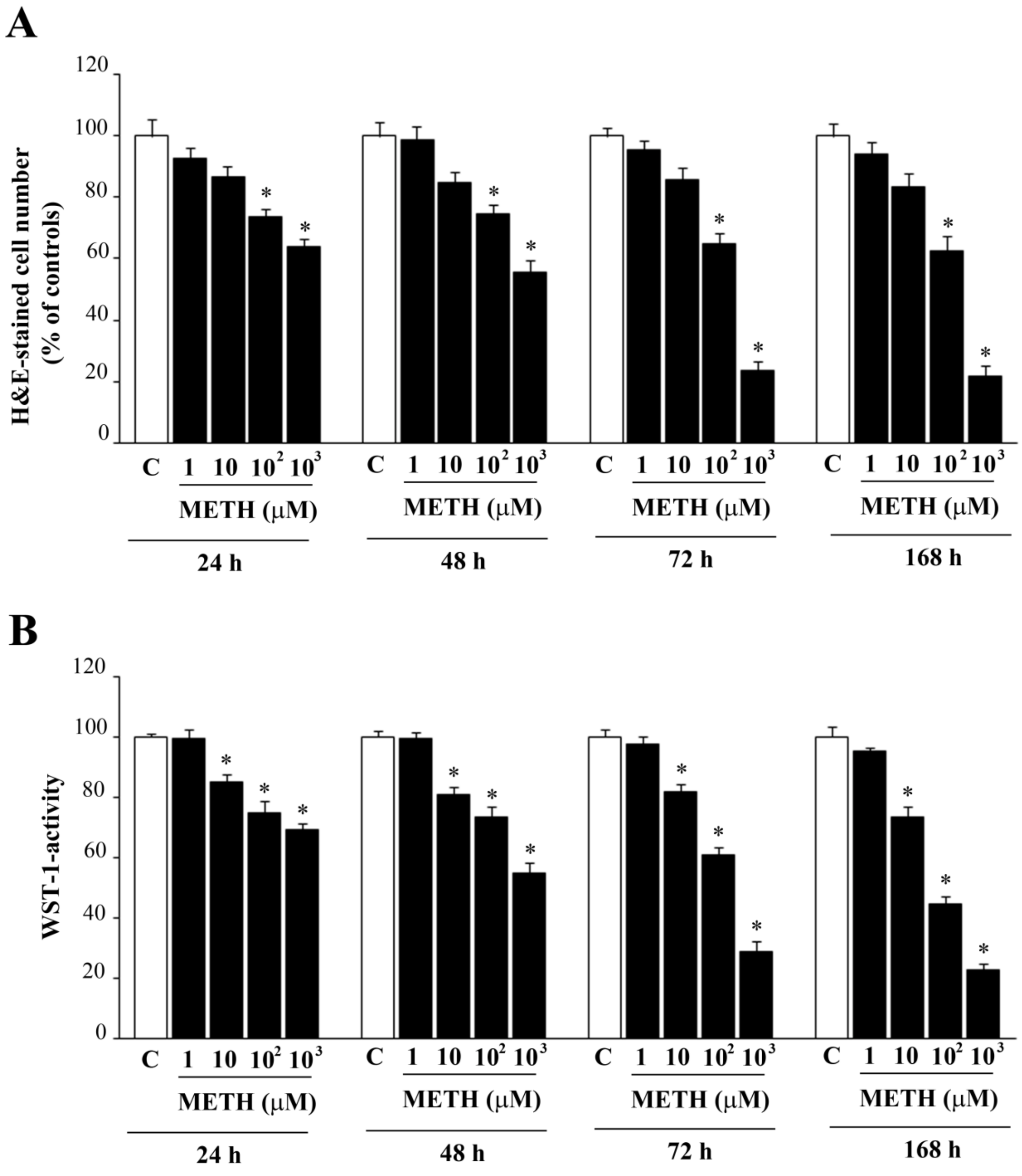

Supplementary Figure 2. Time-dependency of the effects of increasing doses of METH on cell viability. PC12 cell viability after increasing times (from 24 hours up to 168 hours) and doses (from 1 μM up to 1000 μM) of METH, assessed by H&E and WST-1 assay, is reported in the graphs shown in (**A**) and (**B**), respectively. Independently from the time of exposure, METH 100 μM and 1000 μM are toxic for PC12 cells (**A**, **B**), while cell viability is only slightly reduced following METH 10 μM (**B**). Note that the dose-dependent METH toxicity reaches a plateau at 72 hours *p<0.05 compared with control.

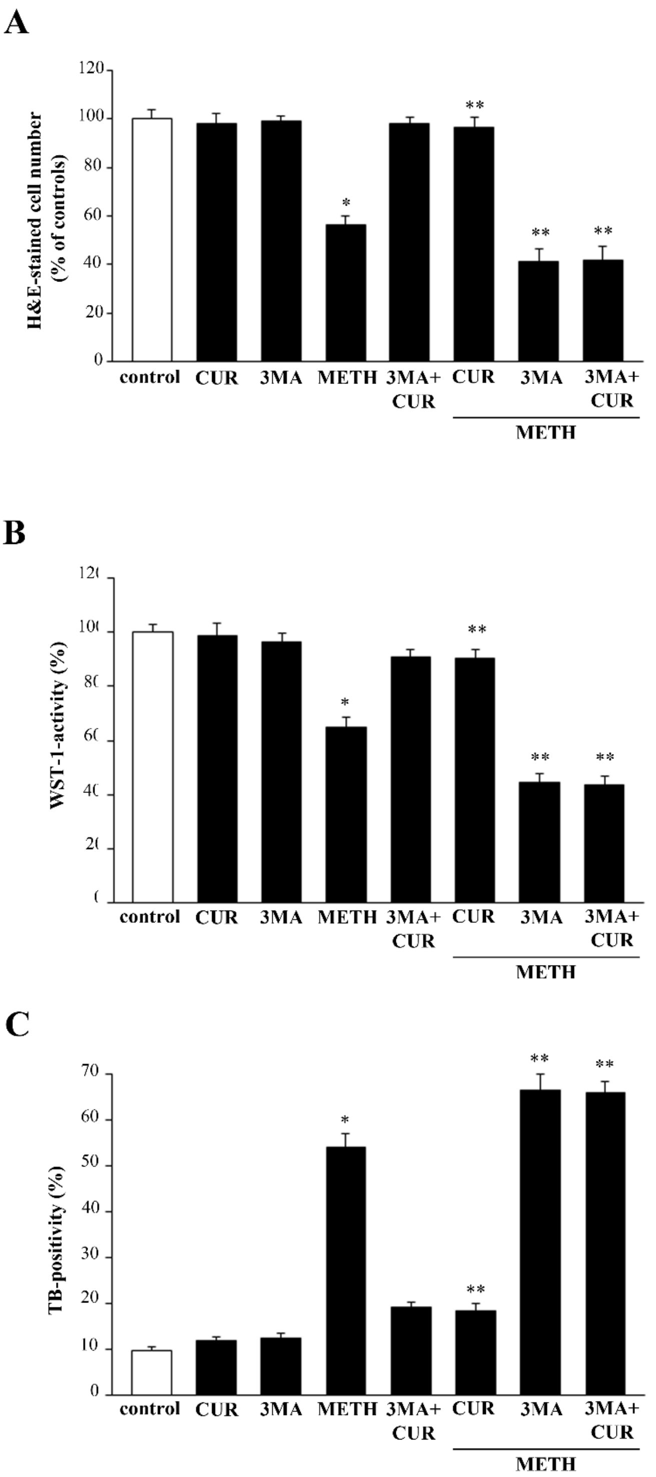

Supplementary Figure 3. Inhibition of autophagy in METH-treated cells induces cell death. PC12 cell survival decreases after 72 hours treatment with the autophagy inhibitor 3MA in the presence of METH (100 μM). Graphs report cell viability measured through H&E staining (**A**), WST-1 activity (**B**), and TB staining (**C**). *p≤ 0.05 compared with control; ** p≤ 0.05 compared with METH.

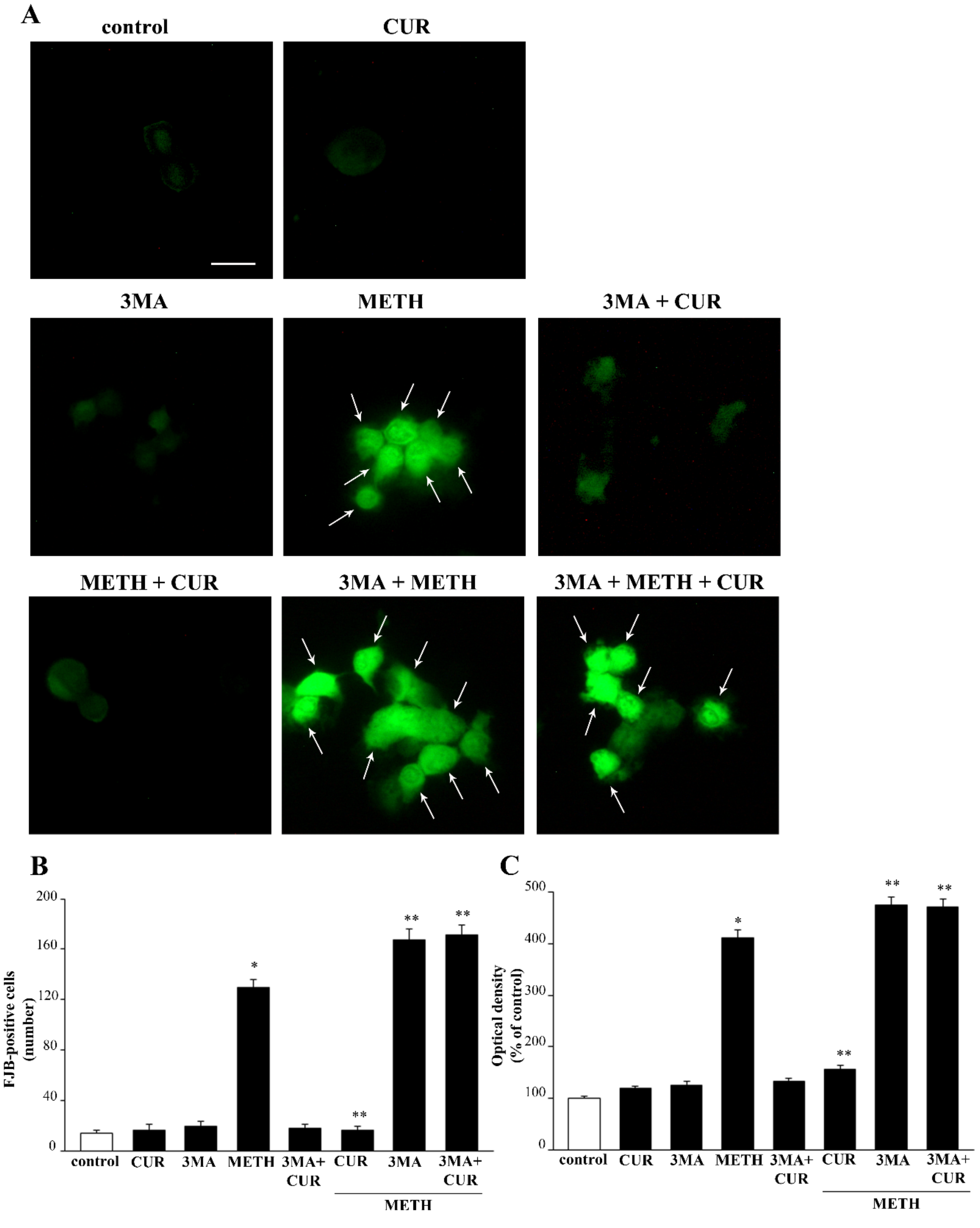

Supplementary Figure 4. Autophagy inhibition increases Fluoro-Jade B-responsiveness of PC12 cells after METH. (**A**) Representative pictures of FJB-stained PC12 cells after 3MA, METH (100 μM), and CUR (10 μM) administration. The graphs show the number (**B**) and the intensity (**C**) of the FJB fluorescent cells. Arrows indicate FJB intensely fluorescent cells.*$p \leq 0.05$ compared with control;**$p < 0.05$ compared with METH. Scale bar = 16 μM.

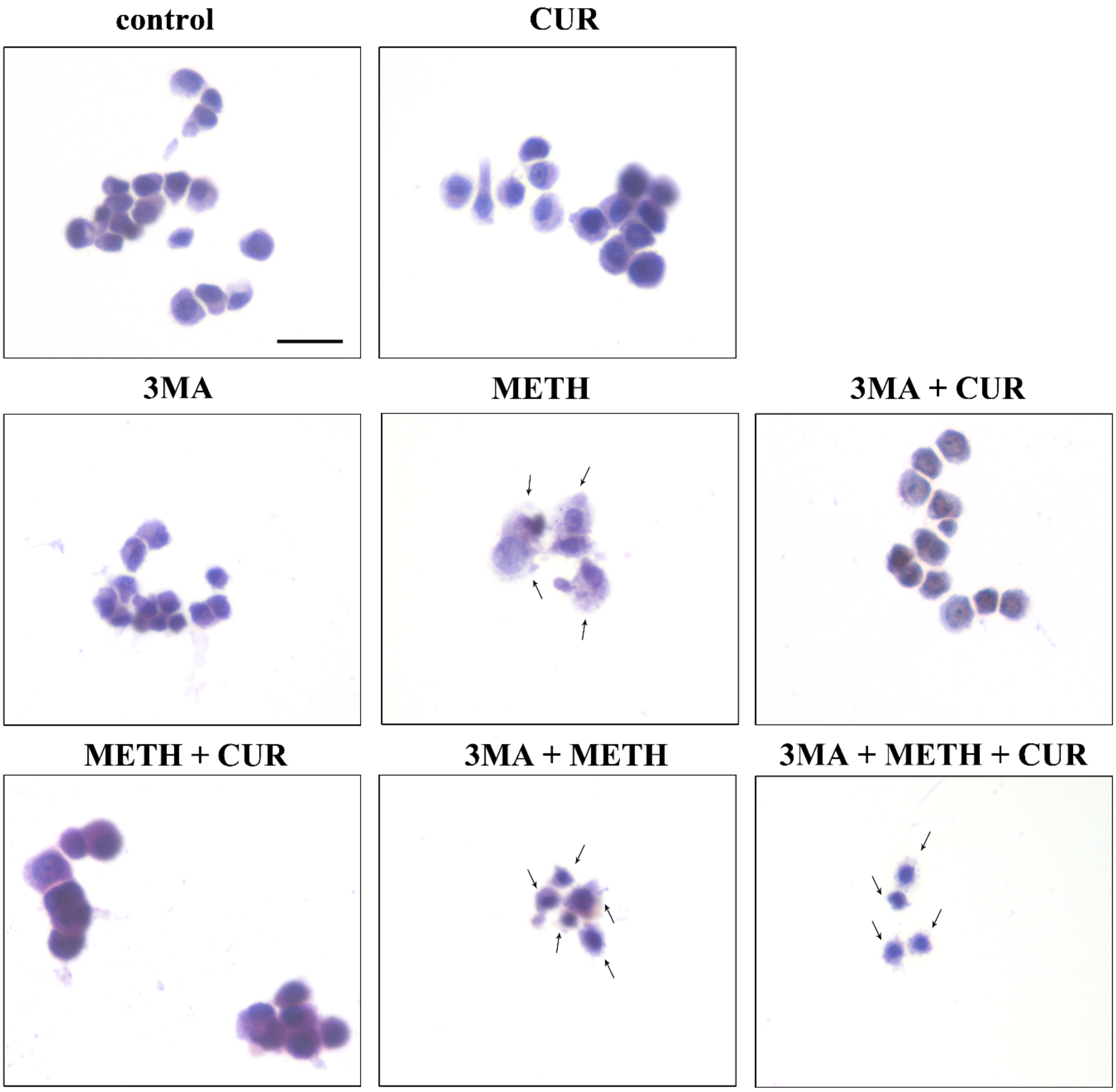

Supplementary Figure 5. Autophagy inhibition increases morphological alterations of PC12 cells after METH. Representative pictures of H&E-stained PC12cells after 3MA, METH (100 µM) and CUR (10 µM) administration for 72 hours. Marked cell alterations (arrows) occur in METH-treated cells pre-administered with 3MA. Scale bar = 26.7 µM.
